# Interventions for pre‐school children in foster care: A systematic review of randomised controlled trials of child‐related outcomes

**DOI:** 10.1002/jcv2.12273

**Published:** 2024-09-05

**Authors:** Natalie Kirby, Camilla Biggs, Megan Garside, Gloria Cheung, Philip Wilson, Matt Forde, Manuela Deidda, Dennis Ougrin, Fiona Turner, Karen Crawford, Helen Minnis

**Affiliations:** ^1^ Leeds and York Partnership NHS Foundation Trust Leeds UK; ^2^ University of Glasgow Glasgow Scotland; ^3^ York and Scarborough Teaching Hospitals NHS Foundation Trust York UK; ^4^ Institute of Applied Health Sciences University of Aberdeen Aberdeen Scotland; ^5^ Division of General Practice Institute for Public Health University of Copenhagen Copenhagen Denmark; ^6^ NSPCC London UK; ^7^ HEHTA School of Health and Wellbeing Glasgow Scotland; ^8^ Youth Resilience Unit Centre for Psychiatry and Mental Health Wolfson Institute of Population Health WHO Collaborating Centre for Mental Health Services Development Queen Mary University of London London UK; ^9^ School of Health and Wellbeing University of Glasgow Glasgow Scotland

**Keywords:** development, foster care, infant, interventions, mental health, pre‐school, relationships

## Abstract

**Background:**

Children in foster care are at high risk of future mental health and developmental difficulties. A number of interventions may be helpful; however, the effectiveness of interventions specifically for pre‐school children in foster care is not well established. This is an important omission, since infancy and early childhood may be the optimal period for interventions to prevent future problems. The current systematic review set out to establish the existing evidence base for interventions to improve social‐emotional, developmental and relational outcomes for pre‐school children in foster and kinship care.

**Methods:**

Searches of online databases were undertaken in June 2023 with keyword search terms related to the study population and design. Studies utilising a randomised control design to measure the effectiveness of interventions for foster children aged 0–7 years were included. The methodological quality of included studies was assessed using the Cochrane Risk of Bias (ROB‐2) tool and effects evaluated using narrative synthesis and GRADE assessments of included interventions and outcomes.

**Results:**

Searches identified 6815 results. Twenty studies, describing seven interventions, met inclusion criteria. Fifteen studies reported intervention benefits comparative to control in at least one outcome domain, with particularly good evidence for Attachment and Behaviour Catch‐Up (ABC) in improving developmental outcomes. There was also evidence for Multi‐Treatment Foster Care for Pre‐Schoolers (MTFC‐P), Kids In Transition To School (KITS), Parent‐Child Interaction Therapy (PCIT) and HeadStart in improving behavioural outcomes. The findings for relational outcomes, including attachment, were mixed; however, there was some evidence for MTFC‐P and ABC in reducing avoidant attachment.

**Conclusions:**

This systematic review contributes to our current understanding of how we might best support pre‐school children in foster care. It remains unclear whether the effectiveness of particular interventions may be moderated by participant or intervention characteristics. Further research is needed to understand which interventions work best for whom in this group. Despite some variability in methodological quality and heterogeneity across studies, our findings suggest that certain interventions are likely to be helpful for young children in foster care. Dissemination and ongoing evaluation of the evidence‐based interventions highlighted within this review should be implemented in clinical practice.

**Key points**

**What's known**

- Research demonstrates that children in foster care are at higher risk of experiencing difficulties in social, emotional, developmental and relational domains. Young children in particular have a high level of need which often goes unmet. Early childhood is a crucial period of development and intervention at this time may be more effective.

**What's new**

- Several interventions including Attachment and Behaviour Catch‐Up (ABC), Multi‐Treatment Foster Care for Pre‐Schoolers (MTFC‐P), Kids in Transition to School (KITS), Parent‐Child Interaction Therapy (PCIT) and HeadStart were found to improve developmental and behavioural outcomes in foster children aged 0–7 years. There was some evidence for MTCF‐P and ABC in reducing avoidant attachment.

**What's relevant**

- We identified an evidence gap for large, high quality trials in this area. Further research is needed to understand how important moderators, such as involvement of birth parents, impact upon child‐related outcomes. Dissemination and ongoing evaluation of the evidence‐based interventions highlighted within this review should be implemented in clinical practice.

## BACKGROUND

Approximately 2.7 million children worldwide are in out of home placements (Petrowski et al., 2017), including approximately 98,000 children in the UK, around 70,000 of whom are living with foster families (Department of Education, 2023). Over 70% of children in foster care have experienced five or more adverse childhood experiences (ACEs) including exposure to violence, substance misuse, and parental mental illness (Bruskas & Tessin, 2013). A range of risk factors often exist before children enter foster care including poverty, parental unemployment and parental relationship breakdown (Jones et al., 2011), in addition to prenatal risk factors such as exposure to alcohol and substances (Smith et al., 2007) and neurodevelopmental conditions such as Attention Deficit Hyperactivity Disorder and Autism (Dinkler et al., 2017). Separation from birth families is traumatic for many children (Mitchell, 2018) and a high proportion experience placement breakdowns resulting in further separations from attachment figures (Ward 2009). This creates a context often characterised by cumulative stress, adversity and trauma (Cloitre et al., 2009; Tarren‐Sweeney, 2023). Children in foster care are at higher risk of experiencing difficulties in social, emotional, developmental and relational domains). This includes higher rates of psychopathology and diagnosed psychiatric conditions (Engler et al., 2022), in addition to difficulties with school adjustment and academic achievement (Fisher, 2015). Looked after young people are five times more likely to die by suicide compared to community controls (Palmer et al., 2021) and are significantly more likely to self‐harm (Lüdtke et al., 2018). A number of studies have reported delays in cognitive development and language abilities (Fisher, 2015, Gyphen 2017), in addition to peer relationships and attachment difficulties (e.g. insecure attachment, attachment disorders) (Engler et al., 2022; Fisher, 2015; Gyphen 2017).

Intervening in early childhood is particularly important, given that the first 1000 days of development (the period between conception and age 2 years) are crucial for subsequent health and well‐being (Moore, 2017). Mental health and developmental problems experienced in the early years can persist into adulthood with potentially lifelong consequences (Lyons‐Ruth et al., 2017, Stewart‐Brown et al., 2005, National Research Council and Institute of Medicine Committee on Integrating the Science of Early Childhood Development, 2000) and interventions are more likely to be effective when offered earlier in life (Heckman & Mosso, 2014). Although children in foster care have a high level of need, these needs often go unmet (Hiller et al., 2022), particularly for children under 3 years (Stahmer et al., 2005).

In recent years, despite methodological challenges, there is a burgeoning evidence base for interventions in foster care. Several reviews (Bergstrom, 2020; Hambrick et al., 2016; Kemmis‐Riggs et al., 2018; Schoemaker et al., 2020; Solomon et al., 2017) have contributed to our understanding of potential benefits of interventions for this group but no review has focussed specifically on interventions for pre‐school children.

### Purpose of review

This systematic review aimed to establish the impact of interventions for pre‐school children in foster care. Although starting‐school age varies between countries, the vast majority have a school‐starting age of six or 7 years (The World Bank, 2024) We therefore included studies involving children aged 0–7 years, in keeping with international conventions for the pre‐school age of children. We refer to children in ‘foster care’ as those living in kinship, defined as care provided by a friend or family member who is not the parent of the child (Foster & Mackley, 2023), or non‐kinship care. We excluded residential settings or group homes as these are likely to involve different needs and resources compared to community settings. Within this paper, we focus on child‐related outcomes, with a separate paper currently underway detailing carer and placement related outcomes. The rationale for this was to ensure that the effects of interventions on children were given sufficient emphasis and understanding in the current paper. We also had a specific aim of examining the extent to which the literature supports/does not support the need for the Best Services Trial (BeST? clinicaltrials.gov identifier NCT01485510)—a randomised controlled trial of a multi‐modal intervention called the New Orleans Intervention Model (NIM) which aims to improve the mental health of children in foster care, their relationships with primary caregivers, and their journeys through the care system. NIM targets the birth family, and often also the foster family, of infants or pre‐school children in foster care. At the initiation of BeST? in 2012 there were few relevant published intervention studies and the recent rapid expansion in the scientific focus on infant mental health necessitates an updated systematic review in this field.

## METHODS

A systematic review was designed in accordance with the Preferred Reporting Items for Systematic Review and Meta‐Analysis (PRISMA) Statement (Moher et al., 2009) and registered on the Prospective Register of Systematic Reviews (PROSPERO) database (CRD 42022367449).

### Search strategy

The purpose of this study was to identify randomised controlled trials (RCTs) of interventions targeting pre‐school children (0–7 years old) in foster care. Preliminary searches used search terms relating to the population of interest (e.g. “looked after children” OR “foster care” OR “foster child” OR “kinship care” OR “safeguarding children”) and filtering for RCTs only.

A broad search strategy included terms as outlined below (see supplementary information for exact search terms).

1. Randomised Controlled Trials, utilising the relevant search filter developed by Scottish Intercollegiate Guidelines Network (Scottish Intercollegiate Guidelines Network (SIGN 2023).
2. Foster care setting, developed through exploration of prior systematic reviews and including relevant items (Turner et al., 2007).
3. Children (0–7 years) utilising search items for Babies, Children and Young People developed by the National Institute of Public Health for Quebec (INSPQ) (Tessier & Lacourse, 2023).

A systematic search of Embase, Medline, CINAHL, PsychInfo and Cochrane Library databases was conducted for all articles published in English before the 23^rd^ June 2023. In an attempt to identify additional published and unpublished studies, we undertook reference searches for all relevant systematic reviews, meta‐analyses and included primary studies. We also used citation searches for included primary studies to identify further papers. In situations where it was not clear whether relevant studies met inclusion criteria, study authors were contacted where possible.

### Eligibility criteria

Studies were selected according to the following inclusion criteria: (a) child participants were under 7 years old, (2) all or almost all (over 80%) of child participants within the study population were living within foster care or kinship care, (3) study data was from an RCT such that an intervention arm was compared with any control condition type (e.g. waitlist, service as usual) and participants had been randomly assigned to the intervention type, (4) full text was available and published in English. Outcomes were intentionally broad in order to reflect that mental health in pre‐school children is multi‐faceted and that various aspects (e.g. behaviour, development, attachment) significantly overlap with one another.

Studies were excluded if the above criteria were not met. This included exclusion of studies where: (a) participant ages ranged beyond 7 years, (2) children were living in residential care, adopted, or living with birth parents (3) only biological outcomes (e.g. cortisol) were reported (4) any non‐RCT study design. We excluded pilot studies if the full RCT had subsequently been published. Please see Table 1 for further information regarding inclusion and exclusion criteria based on the Population, Intervention, Comparators, Outcomes and Study (PICOS) criteria.

**TABLE 1:** PICOS criteria.

	Included	Excluded
**Population**	Children in foster care Children in kinship care Aged 0–7 years	Children in residential settings Children in institutional settings Adopted children Children exposed to or at risk of maltreatment who are not in foster care Aged >7 years
**Intervention**	Psychosocial interventions Interventions can be delivered to foster carers, kinship carers or birth family	No exclusions in terms of setting, format, duration, frequency or intensity of the intervention
**Comparison**	Care as usual/Waitlist control Active control	No exclusions
**Outcomes**	Any measure of child mental health, development, relationship quality or attachment. Including (but not limited to): Child mental health: Psychosocial functioning; internalising symptoms; externalising symptoms; problem behaviours; psychiatric (ICD/DSM) diagnoses Child development: Receptive vocabulary; expressive vocabulary; cognitive functioning; academic attainment; school readiness; self‐regulation; literacy; cognitive flexibility; theory of mind Relationship quality or attachment: Parent‐child interactions; attachment security; attachment disorganisation; diagnoses of attachment disorders	Biological outcomes (e.g. salivary cortisol, heart rate, blood pressure, electrophysiology)
**Study‐type**	Randomised controlled trials (RCTs)	Any non‐RCT study design

### Screening strategy

One researcher (CB) completed title and abstract screening for all identified studies following de‐duplication (*n* = 6815). A second researcher (NK) completed screening for a further 20% of studies, blinded to the first screening outcome. Inter‐rater reliability on this subset of records was 0.98. Discrepancies were discussed and resolved between the two screening researchers. Full texts (*n* = 113) were screened independently by at least two researchers with an inter‐rater reliability of 0.97.

### Data extraction

Four researchers (CB, GC, MG, NK) were involved in the data extraction process, with one researcher (NK) reviewing data extraction for all studies. Data were extracted and tabulated for each study, including study title, author, date of publication, country of research, overall sample demographics, child placement type, sample size overall and for each arm of the RCT, intervention and control characteristics including intensity and duration, outcome measures, follow‐up, confounding variables, and findings. Authors were contacted for further information if required. Duplicates were removed following data extraction (Falconer, 2018).

### Data synthesis

As expected, there was large variation across studies in terms of intervention type and outcomes. As such, pooled statistical analysis of results was not possible. Instead, we performed a narrative synthesis of studies according to intervention type and outcome. Where possible, we calculated standardised effect sizes (Cohen's D) using Wilson's practical Meta‐Analysis Effect Size Calculator (Wilson, 2023) and inputting data using comparisons of mean post‐intervention scores and standard deviations reported in included papers. Where baseline differences in groups were identified, we calculated effect sizes based on pre‐ and post‐test mean scores. An effect size of *d* = 0.20 is considered small, *d* = 0.50 medium, and *d* = 0.80 large (Cohen, 2013). Where relevant data were not available in papers, we indicate this in relevant tables and considered this within risk of bias assessments. Where outcomes were measured at multiple time points, we took a conservative approach and included effect sizes for the longest duration of follow‐up.

### Risk of bias assessment

The Cochrane Risk of Bias 2.0 (RoB2) tool for randomised controlled trials was used to assess the risk of bias for included studies (Sterne et al., 2019). The tool utilises signalling questions within different domains relevant to trial design, conduct and reporting, with each domain contributing to a summary rating of ‘high risk, ‘some concerns’ or ‘low risk’ of bias. Each paper was appraised by two independent researchers. Discrepancies were discussed and resolved, involving a third researcher in resolution if required.

### Grading of evidence

We followed GRADE guidelines (The GRADE Working Group, 2013) to assess the overall quality of evidence for each outcome. All studies were RCTs and therefore had an initial rating of ‘high quality’. Ratings were downgraded as necessary according to the level of concern within each GRADE domain, including; risk of bias, inconsistency, indirectness, imprecision or publication bias. Ratings were downgraded by one level for serious concerns or by two levels for very serious concerns.

### Public involvement

This work was conducted in collaboration with the Best Services Trial (BeST?) team, including the Patient and Public Involvement (PPI) group.

## RESULTS

### Search results

**FIGURE 1:**
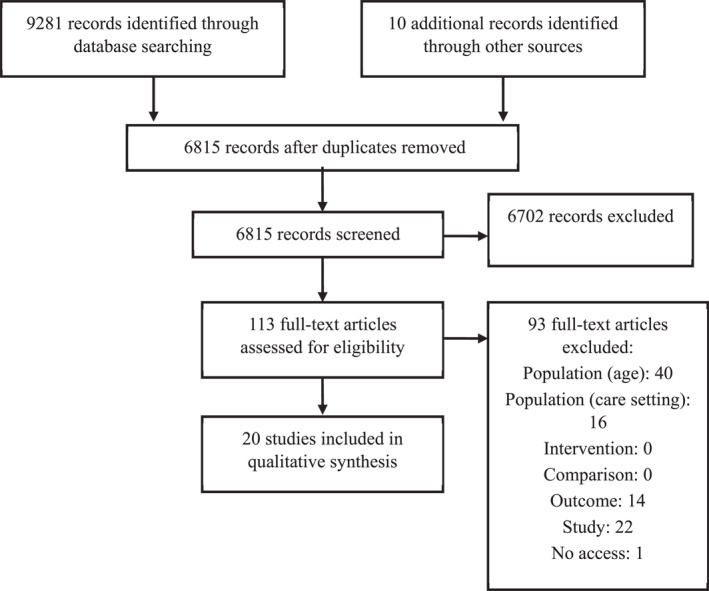
PRISMA flowchart.

Electronic searches produced 6815 papers following de‐duplication. 20 papers were included which evaluated the effectiveness of interventions on mental health, developmental and relational outcomes in children aged 0–7 years in foster and kinship care (see Figure 1 and Table 2 for included studies and PRISMA flowchart).

**TABLE 2:** Summary of included studies.

Study + country	Child age (range)	Sample size	Intervention description	Control group	Main outcome(s)	Outcome timing	Findings
Bernard et al., 2017 USA	34.2–46.4 months	52	**ABC** 10 weekly 1‐h sessions Delivered at participant's home Foster carer and child together Manualised format Delivered by parent trainers	**DEF** 10 weekly 1‐h sessions, 1‐h each, video recorded, at participant's home	Receptive language	2 years post intervention	Higher receptive vocabulary in intervention group compared to control
Dozier et al., 2009 USA	3.9–39.4 months	46			Attachment	1 month post intervention	Reduction in avoidant attachment in intervention group compared to control
Dozier et al., 2006 USA	Child: 3.6–39.4 months	60			Problem behaviours	1 month post intervention	No difference in problem behaviours between groups
Lewis‐Morrarty et al., 2012 USA	4–6 years	61			Theory of mind Receptive language Cognitive flexibility	Annual until the age of 6 years	Higher cognitive flexibility and theory in intervention group compared to control
Lind et al., 2017 USA	14–56 months	173	**ABC‐T** As for ABC Emphasis on co‐regulation		Attention Cognitive flexibility	Post intervention ‐ child aged 48 months	Lower attention difficulties and higher cognitive flexibility in intervention group compared to control
Raby et al., 2019 USA	43.0—61.2 months	88			Receptive vocabulary	Post intervention ‐ child aged between 36 and 60 months old	Higher receptive vocabulary scores in intervention group compared to control
Fisher & Kim, 2007 USA	4.3–4.5 years	117	**MTFC‐P** Duration 6–9 months Delivery separately to foster carers and children Involves birthparents or adoptive families 12 h of foster‐parent training prior to placement + weekly group meetings + phone support Children receive individualised treatment with therapists and weekly therapeutic playgroup session	**Regular foster care** Care as usual. At least monthly contact with caseworkers (may lead to weekly individual psychotherapy, medication or special education services)	Attachment	Post‐intervention at 3‐month intervals up to 12 months	Increase in secure behaviour and decreases in avoidant behaviour in intervention group compared to control
Fisher et al., 2011 USA	4.3–4.5 years	117			Child problem behaviour	Monthly for 3 months	Lower risk of problem behaviours in intervention group compared to control
Bruce et al., 2009 USA	5.51—6.65 years	34			Cognitive control and response monitoring	Not provided	No differences in cognitive control or response monitoring between groups
Jonkman et al., 2017 Netherlands	3—7 years	34		**TAU** 2 weekly home visits ‐ social workers coach foster parents in parental skills and provide children with support. Specific interventions if needed.	Behavioural problems Disturbances of attachment Trauma symptoms	Every 3 months through duration of treatment	No differences in behavioural problems or attachment disorder between groups Improvement in trauma symptoms in control group compared to intervention in last 3 months of intervention
Pears et al., 2012 USA	4.96–5.59 years	192	**KITS** 16‐week group based school readiness curriculum Delivered separately to children + groups for caregivers 24 x 2‐h child sessions twice weekly in the summer + 2‐h sessions once weekly in the fall 8 x 2‐h caregiver groups fortnightly School readiness phase + transition/maintenance phase	**Foster care comparison** Service as usual	Oppositional and aggressive classroom behaviours	Up to 8 months post‐intervention	Reduced oppositional and aggressive behaviours in intervention group compared to control
Pears et al., 2013 USA	4.96–5.59 years	192			Early literacy skills Prosocial skills Emotional regulation	4 months post‐intervention	Improved early literacy and self‐regulatory skills in intervention group compared to control No differences in prosocial skills
Pears et al., 2016 USA	4.96–5.59 years	192			Positive attitudes towards alcohol and antisocial behaviour Involvement with deviant peers Self‐competence	Post intervention age 5 + 6 years + subsequent school years up to 9 years old	Decreased positive attitudes towards alcohol use and antisocial behaviours and increased self‐competency in intervention group compared to control
Mersky et al., 2016 USA	3—6 years	102	**PCIT** Delivered to carer and child together Individual (typically 12–20 sessions) + group foster parent training Brief—2xdays training, 8 weeks phone calls Extended—2xdays training, 14 weeks phone calls	**Waitlist control** Services as usual	Eyberg child behaviour inventory Child behaviour checklist	8 + 14 weeks	Reduction in externalizing and internalizing scores for both intervention groups compared to control at 8 weeks and in favour of extended PCIT at 14 weeks
Danko, 2014 USA	2.57—4.55 years	24	**CDI only** 10‐14 × 60‐min sessions twice a week **CDI plus PDI** 5‐7xCDI and 5‐7xPDI sessions twice a week 10‐14 sessions Therapist coaching, feedback + homework Delivered to carer and child together	**Waitlist bibliotherapy** Educational handouts with written description PCIT skills + additional parenting “tips sheets”	Child behaviour Attachment	Immediately post intervention	Reduced behavioural difficulties in both intervention groups compared to control No significant difference in attachment between groups
N'zi et al., 2016 USA	2.0–7.5 years	14	**CDI** 8xtwice weekly sessions Therapist coaching, feedback + homework Delivered to carer and child together	**Waitlist control** Service as usual	Child behaviour Child‐parent relationship	7 weeks post baseline 3 months post intervention (for CDIT group only)	Improvement in relationship quality and reduction in behavioural problems in intervention group compared to controls
Conn et al., 2018 USA	36.52—70.14 months	33	**IY** 2.5‐h weekly sessions, weekly for 13 weeks Delivered to caregivers in group format Community‐based location Parent groups led by masters‐level psychologist certified in IY curriculum	**TAU**	Child behaviour	Post intervention 13 weeks	No between group differences were found for measures of child behaviour.
Job et al., 2022 Germany	24–91 months	87	**TCTP** Delivered to foster carers Five 2.5‐h weekly group sessions + two 20‐min telephone consultations Intervention mean length of 7.2 weeks	**Usual care**	Carer‐child relationship and interaction Child behaviour Child anxiety	6–12 months post baseline	No differences between groups in relationship quality, child behaviour and anxiety
Lipscomb et al., 2013 USA	41.55—54.95 months	253	**HeadStart** Publicly financed early childhood education and care programme Comprehensive services to support disadvantaged preschool‐age children and their families Provides quality early learning, parental support, and wrap‐around services	**Community control group** Could be enroled in HeadStart in the second year of the study	Teacher‐child relationship Behaviour problems Pre‐school academic skills	At the end of the HeadStart year +1 year later	Increased pre‐academic skills, more positive teacher–child relationships, and reductions in behaviour problems in intervention group compared to control at post‐intervention; indirect effects (mediated by earlier effects) noted 12 months later
Kyunghee & Jung‐Sook, 2016 USA	2.9—3.9 years	162		**Non‐HeadStart group** Could be enroled in HeadStart in the second year of the study if they remained eligible and did not go on to kindergarten	Child and parent relationship Child and teacher relationship Hyperactivity and aggression Social skills and positive approaches to learning	At the end of the HeadStart year + 1 year later	Lower hyperactivity scores and more positive attitudes to learning in intervention group compared to control

Abbreviations: ABC, Attachment and Biobehavioural Catch‐Up; DEF, Developmental Education for Families; ABC‐T, Attachment and Biobehavioural Catch‐Up for Toddlers; MTFC‐P, Multidimensional Treatment Foster Care for Preschoolers; TAU, Treatment as usual; KITS, Kids in Transition to School; PCIT, Parent Child Interaction Therapy; CDI, Child‐Directed Interaction; PDI, Parent‐Directed Interaction; IY, Incredible Years; TCTP, Triple P system for foster parents.

#### Study design and characteristics

All 20 studies used a randomised controlled trial study design. Eighteen studies were based in the USA and two in Europe, in the Netherlands (Jonkman et al., 2017) and Germany (Job et al., 2022). Eighteen studies compared one intervention group against a control, while two compared two intervention groups against a control group (Danko, 2014; Mersky et al., 2016). See Table 2 for summary of included studies and supplementary information for further detail of study characteristics.

#### Participants

Sample sizes ranged from 14 (N'zi et al., 2016) to 253 (Lipscomb et al., 2013), with a total of 1460 children aged 18 months (Dozier et al., 2009) to 6 years (Bruce et al., 2009). Most interventions targeted children aged between 2 and 7 years.

Thirteen studies were conducted with foster carers (Bernard et al., 2017; Bruce et al., 2009; Conn et al., 2018; Dozier et al., 2006, 2009; Fisher et al., 2011; Fisher & Kim, 2007; Job et al., 2022; Jonkman et al., 2017; Lewis‐Morrarty et al., 2012; Lind et al., 2017; Mersky et al., 2016; Raby et al., 2019). The remaining studies involved a mixture of foster carers and kinship carers (Danko, 2014; Pears et al., 2012, 2013, 2016), ‘non‐parental’ carers (primary caregivers who self‐identify as someone other than a biological, adoptive, or step‐parent) (Kyunghee & Jung‐Sook, 2016; Lipscomb et al., 2013) and kinship carers only (N'zi et al., 2016). One RCT (Bruce et al., 2009; Fisher et al., 2011; Fisher & Kim, 2007) also involved ‘permanent placement resources’ (i.e. birth parents, adoptive relatives, and non‐relatives) in the intervention where possible.

#### Interventions and controls

Seven interventions were included across studies; Attachment and Behaviour Catch‐Up (ABC) (Bernard et al., 2017; Dozier et al., 2009; Lewis‐Morrarty et al., 2012) including ABC‐Toddler (ABC‐T) (Lind et al., 2017; Raby et al., 2019), Multi‐Treatment Foster Care for Pre‐Schoolers (MTFC‐P) (Bruce et al., 2009; Fisher et al., 2011; Fisher & Kim, 2007; Jonkman et al., 2017), Kids In Transition to School (KITS) (Pears et al., 2012, 2013, 2016), Parent Child Interaction Therapy (PCIT) (Danko, 2014; Mersky et al., 2016; N'zi et al., 2016), Incredible Years (IY) (Conn et al., 2018), Taking Care Triple P (TCTP) (Job et al., 2022) and HeadStart (Kyunghee & Jung‐Sook, 2016; Lipscomb et al., 2013). Of these interventions, five were evaluated within a single trial, and three were assessed in two or more trials.

Eleven studies delivered interventions to foster carers and children together (Bernard et al., 2017; Conn et al., 2018; Danko, 2014; Dozier et al., 2009; Dozier et al., 2006; Kyunghee & Jung‐Sook, 2016; Lewis‐Morrarty et al., 2012; Lind et al., 2017; Lipscomb et al., 2013; Mersky et al., 2016; N'zi et al., 2016; Raby et al., 2019). Seven studies (Bruce et al., 2009; Fisher et al., 2011; Fisher & Kim, 2007; Jonkman et al., 2017; Pears et al., 2012, 2013, 2016, 2016, 2016) delivered the intervention separately to carers and children, and two were delivered to carer only (Conn et al., 2018; Job et al., 2022).

Seven studies used a dummy intervention as a control condition (Bernard et al., 2017; Danko, 2014; Dozier et al., 2009; Lewis‐Morrarty et al., 2012; Lind et al., 2017; Raby et al., 2019) with the remainder using foster care services as usual, including waitlist controls.

No studies specifically targeted birth families of children already placed in foster care.

#### Outcomes and measures

Child behavioural outcomes were assessed within 12 studies (Conn et al., 2018; Danko, 2014; Dozier et al., 2006; Fisher et al., 2011; Job et al., 2022; Jonkman et al., 2017; Kyunghee & Jung‐Sook, 2016; Lipscomb et al., 2013; Mersky et al., 2016; N'zi et al., 2016; Pears et al., 2013; Pears et al., 2012). Studies used continuous measures such as the Child Behaviour Checklist (CBCL), Child Behaviour Questionnaire (CBQ), Eyberg Child Behaviour Inventory (ECBI) or Parent Daily Report (PDR) allowing a comparison of mean scores in relevant domains (e.g. ‘problem behaviour’ scores) for treatment and control groups.

Eight studies measured children's academic or developmental outcomes (Bernard et al., 2017; Bruce et al., 2009; Lewis‐Morrarty et al., 2012; Lind et al., 2017; Lipscomb et al., 2013; Pears et al., 2013, 2016; Raby et al., 2019). These included measures of the child's cognitive skills including theory of mind (Penny Hiding Task), cognitive flexibility (Dimensional Change Card Sort), regulatory skills (Emotion Regulation Checklist), and measures of language and vocabulary (Peabody Vocabulary Test, Dynamic Indicators of Basic Early Literacy Skills).

Eight studies included outcomes relating to attachment and parent‐child relationships (Danko, 2014; Dozier et al., 2009; Fisher & Kim, 2007; Jonkman et al., 2017; Kyunghee & Jung‐Sook, 2016; N'zi et al., 2016; Raby et al., 2019). Measures included; Attachment Q‐Set, Disturbances of Attachment Interview, Child‐Parent Relationship Scale and Parent Attachment Diary. Some studies used observations such as the Strange Situation Procedure.

#### Follow‐up

The duration of follow‐up varied across the studies. Three studies measured immediate post‐intervention outcomes (Danko, 2014; N'zi et al., 2016; Pears et al., 2013). Nine studies measured outcomes up to 12 months with the remainder measuring outcomes up to 18 months (Lind et al., 2017), 2 years (Bernard et al., 2017; Kyunghee & Jung‐Sook, 2016; Lewis‐Morrarty et al., 2012), 3 years (Raby et al., 2019), and 4 years post‐intervention (Pears et al., 2016).

### Risk of bias

The 20 studies were characterised by significant methodological heterogeneity including differences in intervention duration and intensity, setting, length of follow‐up and outcome measures. The methodological quality of the included studies was variable; none of the studies were rated as having an overall low risk of bias (see Table 3). As is often the case for psychosocial interventions (Juul et al., 2021), 13 studies were rated as being at high risk of bias due to lack of blinding of participants and intervention providers, with the remainder given a rating of moderate risk of bias in this domain. More weighting was given to the potential effects of a lack of blinding of participants where studies did not include multi‐informant outcomes (for example, where only carer‐rated outcomes were utilised). The majority of studies did not comment specifically on blinding and most did not discuss the potential implications of a lack of blinding, for example, in terms of performance and detection bias. Overall, there was also a lack of sufficient information regarding methods for random sequence generation or allocation concealment, leading to an unclear risk of bias for the majority of studies in this domain. There were also concerns regarding attrition, inappropriate statistical analyses (i.e. lack of intention to treat analyses) and/or management of missing outcome data in a number of studies.

**TABLE 3:** Risk of bias summary.

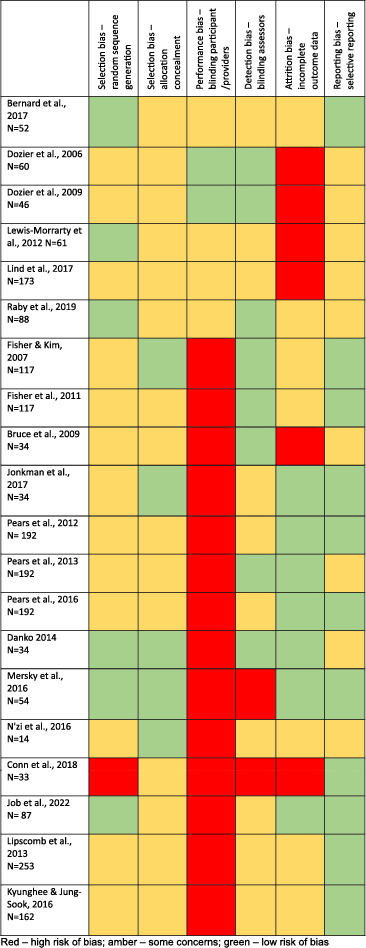

## FINDINGS

### Narrative summary of findings according to intervention

#### ABC and ABC‐T interventions

See Table 4 for a summary of findings for each intervention.

**TABLE 4:** Summary of findings by intervention.

	Intervention focus/framework	Study	Control	Follow up	Social and emotional wellbeing	Development/academic	Relational	Measures	Cohen's *D* (95% CI)
**ABC**	Caregiver sensitivity, responsiveness, nurturance Attachment framework	Bernard et al., 2017 *N* = 52	DEF	2 years	‐	1) Receptive vocabulary	‐	PPVT (3^rd^ edition)	1) 0.65 (0.09, 1.21)
		Dozier et al., 2006 *N* = 60	DEF	1 month	*Behaviour problems*	‐	‐	PDR	‐
		Dozier et al., 2009 *N* = 46	DEF	1 month	‐	‐	1) Avoidant attachment *Secure attachment*	PAD SSP	1) −0.68 (−1.27,‐0.08)
		Lewis‐Morrarty et al., 2012 *N* = 61	DEF	2 years	‐	1) Cognitive flexibility 2) Theory of mind	‐	DCCS Penny hiding game	1) 1.03 (0.34, 1.72) 2) 1.08 (1.04, 1.36)
**ABC‐T**		Lind et al., 2017 *N* = 173	DEF	18 months	‐	1) Cognitive flexibility 2) Attention regulation	‐	DCCS CBCL (attention problems scale)	1) 0.40 (0.04, 0.76) 2) −0.42 (−0.79, −0.06)
		Raby et al., 2019 *N* = 88	DEF	3 years	‐	1) Receptive vocabulary	‐	PPVT (3rd edition)	1) 0.44 (0.02, 0.86)
**MTFC‐P**	Child self‐regulation, caregiver responsiveness and consistency Attachment framework Dyadic intervention	Fisher & Kim, 2007 *N* = 117	RFC	12 months	‐	‐	1) Secure attachment 2) Avoidant attachment *Resistant attachment*	PAD	1) 0.48 (0.11, 0.85) 2) −0.60 (−0.97, −0.23)
		Fisher et al., 2011 *N* = 117	RFC	3 months	1) Behavioural difficulties	‐	‐	PDR	1)^a^
		Bruce et al., 2009 *N* = 34	RFC	Not stated	‐	*Cognitive control* *Response monitoring*	‐	Flanker task	‐
		Jonkman et al., 2017 *N* = 34	TAU	9 months	*Problem behaviours* *Trauma symptoms*	‐	*Disturbance in attachment*	CBCL PDR TRF DAI TSYTC	‐
**KITS**	Child self‐regulation, literacy, prosocial skills Social learning framework	Pears et al., 2012 *N* = 192	FCC	8 months	1) Oppositional/aggressive behaviour	‐	‐	TRF ‐aggressive + delinquent subscales Conners (teacher) ‐ oppositional subscale	1) −0.33 (−0.61, −0.04)
		Pears et al., 2013 *N* = 192	FCC	4 months	*Prosocial behaviour*	1) Literacy 2) Regulatory skills	‐	DIEBELS Concepts of print test Caregiver rating of prereading skills PIPPS CBQ BRIEF‐P CBCL ERC	1) 0.26^+^ 2) 0.18^+^
		Pears et al., 2016 *N* = 192	FCC	4 years	‐	1) Self‐competence 2) Attitudes to alcohol *Attitudes to antisocial behaviour* *Involvement with deviant peers*	‐	Monitoring the future national survey questions Positive attitudes towards antisocial behaviour/deviant peer involvement (bespoke) SPCC	1) 0.50 (0.21, 0.79) 2) −0.42 (−0.71, −0.14)
**PCIT**	Parenting skills, reflection, nurturance, child behaviour Attachment/social‐learning framework Dyadic behavioural intervention	Danko, 2014 *N* = 34	Group 1: Waitlist biblio‐therapy Group 2: CDI‐only Group 3: Full PCIT (CDI + PDI)	Immediate	1) Behavioural difficulties (G1 vs. *G*2+3)	‐	*Secure attachment*	ECBI CBCL Attachment Q‐Set	1) 0.70 (0.009, 1.39)
		Mersky et al., 2016 *N* = 54	Group 0: Waitlist control Group 1: Brief PCIT Group 2: Extended PCIT	14 weeks	1) Behavioural difficulties (G0 vs. G2) 2) Behavioural difficulties (G1 vs. G2) *Behavioural difficulties (G0* vs. *G1)*	‐	‐	ECBI CBCL	1) 1.15 (0.47, 1.83) 2) 0.71 (0.05, 1.37) (in favour of G2)
		N'zi et al., 2016 *N* = 14	Waitlist control	3 months	1) Externalising behaviour *Internalising behaviour*	‐	2) Child carer relationship quality	ECBI CBCL PDR DPICS CPRS	1) 1.26 (0.15, 2.37) 2) 1.24 (0.10, 2.38)
**IY**	Parenting skills, responsiveness, nurturance Attachment/social‐learning framework	Conn et al., 2018 *N* = 33	TAU	12 months	*Behaviour problems* *Externalising symptoms* *Internalising symptoms*	‐	‐	CBCL	‐
**TCTP**	Positive parenting, child behaviour Social learning framework	Job et al., 2022 *N* = 87	TAU	12 months	*Behaviour problems* *Anxiety* *ICD‐10 diagnoses*	‐	*Child‐carer relationship*	ECBI PAS PCPTOS, 4^th^ edition DICA PCRI DPCIS	‐
**Head start**	Child health, development and learning Developmental framework	Lipscomb et al., 2013 *N* = 253	CC	1 year post‐baseline	1) Behaviour problems	2) Academic skills	3) Teacher child relationship	CBCL STRS ASPI	1) −0.40 (−0.03, −0.76) 2) 0.33 (0.05, 0.62) 3) 0.64 (0.27, 1.02)
		Kyunghee & Jung‐Sook, 2016 *N* = 162	CC	2 years	1) Hyperactivity *Aggression* *Social skills* *Attitudes to learning*		*Teacher‐child relationship* *Parent‐child relationship*	ASPI Parent‐rated social skills/positive approaches to learning robert pianta scale ‐ total positive relationship scale, positive child and teacher relationship	1) −0.38 (−0.03, −0.07)

^a^ Unable to calculate effect size from information in paper; + Unable to calculate confidence intervals from information in paper. Statistically significant (*p* < .05) results shown in red; non‐significant effects shown in italics.

Abbreviations: ABC; Attachment and Biobehavioural Catch‐Up; DEF, Developmental Education for Families PPVT, Peabody Picture Vocabulary Test; PDR, Parent Daily Report; PAD, Parent Attachment Diary; SSP, Strange Situation Procedure; ABC‐T, Attachment and Biobehavioural Catch‐Up for Toddlers; CBCL, Child Behaviour Checklist; MTFC‐P, Multidimensional Treatment Foster Care for Preschoolers; TAU, Treatment as usual; DAI, Disturbances of Attachment Interview; TSYTC, Trauma Symptom Checklist for Young Children; TRF, Teacher Report‐Form; KITS, Kids in Transition to School; DIEBELS, Dynamic Indicators of Basic Early Literacy Skills; PIPPS, Preschool Penn Interactive Peer Play Scale; CBQ, Children's Behaviour Questionnaire; BRIEF‐P, Brief Rating Inventory of Executive Function–Preschool Version; ERC, Emotion Regulation Checklist; SPPC, Self‐Perception Profile for Children; ECBI, Eyberg Child Behaviour Inventory; PCIT, Parent Child Interaction Therapy; CDI, Child‐Directed Interaction; PDI, Parent‐Directed Interaction; IY, Incredible Years; TCTP, Triple P system for foster parents; DPCIS, Dyadic Parent‐Child Interaction Coding System; CPRS, Child‐Parent Relationship Scale; CRDI, Child Relationship Development Inventory; PAS, Preschool Anxiety Scale; PCPTOS, Parent–Child Play Task Observation System; DICA, Diagnostic Interview of Mental Disorders in Childhood and Adolescents; STRS, Student–Teacher Relationship Scale; ASPI, Adjustment Scales for Preschool Intervention; FCC, Foster Care Control; CC, Community Care.

Four papers from two RCTs assessed the impact of the ABC intervention (Bernard et al., 2017; Dozier et al., 2006, 2009; Lewis‐Morrarty et al., 2012) with two papers from a third RCT looking specifically at ABC‐T (Lind et al., 2017; Raby et al., 2019). All used a control (Developmental Education for Families) as the comparison intervention. ABC is a brief home‐visiting intervention involving ten 1‐h weekly sessions focussed on improving parental sensitivity, initially developed for infants and subsequently adapted for toddlers (Dozier et al., 2014). Of the included studies, four reported that the ABC intervention improved child developmental outcomes and one study found that ABC led to reductions in avoidant attachment.

#### MTFC‐P intervention

Four studies measured the effect of the MTFC‐P intervention (also known as Early Intervention in Foster Care). MTFC‐P is a 6–9 months intensive multi‐component programme that incorporates foster carer training, child behavioural therapy and involvement of long‐term placement resources for example, birth and adoptive families (Fisher et al., 1999). Three papers linked to the same RCT compared MTFC‐P to regular foster care in children aged 3–6 years (Bruce et al., 2009; Fisher et al., 2011; Fisher & Kim, 2007). One study reported reductions in avoidant attachment and modest improvements in secure attachment (Fisher & Kim, 2007), while a second found no evidence of reductions child behavioural problems or attachment disorder behaviours (Jonkman et al., 2017). A further study found no evidence of improvements in child cognitive outcomes (Bruce et al., 2009) but did report an overall treatment effect in favour of treatment as usual in comparison to MTFC‐P for child trauma symptoms.

#### KITS intervention

Three studies, linked to the same RCT, studied the KITS intervention in children aged 5 years (Pears et al., 2012, 2013, 2016). KITS is a short‐term intensive intervention consisting of therapeutic child playgroups, alongside parent support groups, focussed on building skills necessary for school adjustment. The studies reported positive changes in child literacy and regulatory skills (Pears et al., 2012) and child oppositional/aggressive behaviours (Pears et al., 2013). They also found evidence of longer‐term beneficial effects up to 4 years following KITS (Pears et al., 2016).

#### PCIT intervention

Three RCTs measured the impact of PCIT (Danko, 2014; Mersky et al., 2016; N'zi et al., 2016). PCIT is a dyadic intervention conducted through coaching sessions consisting of two phases: Child‐Directed Interaction (CDI) with a focus on building positive carer‐child relationships; and Parent‐Directed Interaction (PDI), focussing on specific techniques to manage child behaviours (Hembree‐Kigin & McNeil, 2013). Danko, 2014 demonstrated marginally significant reductions in behavioural problems but no differences in attachment security. N'zi et al., 2016 reported more positive relationships and fewer externalizing child behaviour problems and Mersky et al., 2016 also reported reductions in child behaviour problems following PCIT.

#### IY intervention

One pilot RCT (Conn et al., 2018) evaluated a trauma‐informed version of IY, involving a 14‐week parent group intervention focussed on positive parenting skills using social learning and behavioural principles (Webster‐Stratton & Reid, 2010). The authors reported an improvement in behavioural outcomes, but this did not reach statistical significance.

#### Triple P intervention

One RCT evaluated a short‐term version of Triple P comprising weekly parenting groups and telephone support, focussed on positive parenting and behaviour management, in children aged 2–7 years (Job et al., 2022). The study found no significant differences in terms of child behaviour, anxiety, child‐carer relationships and interactions compared to care as usual.

#### HeadStart

Two studies measured the impact of the HeadStart intervention in children aged 4 years using a subsample of children from a larger RCT, the US HeadStart Impact Study (HSIS). HeadStart is a federally funded comprehensive programme for low‐income families based on a ‘whole child’ approach, involving education, medical, dental and mental health, with a focus on family engagement (Services, 2010; US Department of Health and Human Services, 2010). One study (Lipscomb et al., 2013) reported significant impacts of HeadStart on children's pre‐academic skills and relationships with preschool teachers, as well as child behavioural problems, improved academic, relational and behavioural outcomes. A second paper (Kyunghee & Jung‐Sook, 2016) found evidence of longer term effects, reporting that children who had participated in HeadStart had lower hyperactivity scores compared to a community control group.

### GRADE assessments

#### Child social and emotional outcomes

Child social and emotional outcomes were assessed in 12 studies, including 1040 children, with follow‐up between 0 and 24 months. Overall, seven studies (826 participants) found evidence of intervention effects in this domain, with six studies demonstrating effect sizes above 0.20.

Twelve studies measured child behaviours, seven of which reported significant effects on externalising, internalising and overall problem behaviours. Six studies, involving MTFC‐P, KITS, PCIT and HeadStart, reported a standard mean difference (SMD) above 0.20 in at least one domain of child behaviour (Kyunghee & Jung‐Sook, 2016; Lipscomb et al., 2013; Mersky et al., 2016; N'zi et al., 2016; Pears et al., 2012). For the remaining study (Fisher et al., 2011), effect sizes could not be calculated due to relevant data not being available.

Two studies, evaluating TCTP and MTFC‐P, used specific child mental health outcome measures (Job et al., 2022; Jonkman et al., 2017) neither of which found evidence of positive intervention effects for child anxiety, trauma symptoms or ICD‐10 diagnoses.

Two studies, involving KITS and HeadStart, measured child prosocial behaviour (Pears et al., 2016) and social skills (Kyunghee & Jung‐Sook, 2016). Neither found evidence of improvements in these domains following the intervention.

The quality of the evidence for child social and emotional outcomes was downgraded due to the risk of bias (−1) and imprecision (−1) (see Table 5). There was a high or unclear risk of bias in nine studies due to lack of blinding of participants or intervention providers. Two studies were rated as high risk for possible attrition bias due to a lack of information regarding loss to follow‐up and statistical methods to correct for missing data. One study did not use intention to treat analyses. At least 12 different measures were used across studies; the Child Behaviour Checklist and Eyberg Child Behaviour Inventory were used most often.

**TABLE 5:** GRADE assessments.

	Number of studies Participants	Follow‐up	Results	Effect sizes	Interventions with demonstrated ES > 0.20	Grading strength
**Social and emotional outcomes**	12 studies 1040 participants	0–24m	Comparative benefit of intervention demonstrated in 7/12 studies (826 participants)	Effect sizes > 0.20 demonstrated in 6 studies in at least one domain	HeadStart PCIT KITS MTFC‐P	**Low** Downgraded due to risk of bias (−1)^a^ and inconsistency^b^ (−1)
Internalising behaviour	2 studies 47 participants	Immediate—12m	Comparative benefit of intervention demonstrated in 0/2 studies	Effect sizes > 0.20 demonstrated in 0/2 studies	n/a	‐
Externalising behaviour	4 studies 401 participants	Immediate—2 years	Comparative benefit of intervention demonstrated in 3/4 studies	Effect sizes > 0.20 demonstrated in 3/4 studies	HeadStart PCIT KITS	‐
Problem behaviour/behavioural difficulties	8 studies 864 participants	Immediate—12m	Comparative benefit of intervention demonstrated in 4/8 studies	Effect sizes > 0.20 demonstrated in 3/8 studies	HeadStart PCIT MTFC‐P	‐
Social: social skills, prosocial behaviour	2 studies 354 participants	4m—2years	Comparative benefit of intervention demonstrated in 0/2 studies	Effect sizes > 0.20 demonstrated in 0/2 studies	‐	‐
Specific mental health: anxiety, trauma symptoms, ICD‐10 diagnoses	2 studies 121 participants	9—12m	Comparative benefit of intervention demonstrated in 0/2 studies	Effect sizes > 0.20 demonstrated in 0/2 studies	‐	‐
**Developmental outcomes**	8 studies 1015 participants	4m—4years	Better outcomes for intervention group demonstrated in 7/8 studies (981 participants)	Effect sizes > 0.20 demonstrated in 7 studies in at least one domain	ABC	**Moderate** Downgraded due to risk of bias^a^ (−1)
Receptive vocabulary	2 studies 140 participants	2‐3 years	Comparative benefit of intervention demonstrated in 2/2 studies	Effect sizes > 0.20 demonstrated in 2/2 studies	ABC	‐
Cognitive flexibility	2 studies 234 participants	18m—2 years	Comparative benefit of intervention demonstrated in 2/2 studies	Effect sizes > 0.20 demonstrated in 2/2 studies	ABC	‐
**Relational outcomes**	8 studies 585 participants	0–12m	Better outcomes demonstrated for intervention group demonstrated in 4/8 studies (430 participants)	Effect sizes > 0.20 demonstrated in 4 studies in at least one domain	ABC MTFC‐P PCIT HeadStart	**Low** Downgraded due to risk of bias^a^ (−1) and inconsistency^c^ (−1)
Secure attachment	3 studies 197 participants	Immediate—12m	Comparative benefit of intervention demonstrated in 1/3 studies	Effect sizes > 0.20 demonstrated in 0/3 studies	n/a	‐
Avoidant attachment	2 studies 163 participants	1—12m	Comparative benefit of intervention demonstrated in 2/2 studies	Effect sizes > 0.20 demonstrated in 2/2 studies	ABC MTFC‐P	‐
Carer‐child relationship	3 studies 263 participants	Immediate—2 years	Comparative benefit of intervention demonstrated in 1/3 studies	Effect sizes > 0.20 demonstrated in 1/3 studies	PCIT	‐

Abbreviations: ABC, Attachment and Biobehavioural Catch‐Up; DEF, Developmental Education for Families; ABC‐T, Attachment and Biobehavioural Catch‐Up for Toddlers; MTFC‐P, Multidimensional Treatment Foster Care for Preschoolers; TAU, Treatment as usual; KITS, Kids in Transition to School; PCIT, Parent Child Interaction Therapy; CDI, Child‐Directed Interaction; PDI, Parent‐Directed Interaction; IY, Incredible Years; TCTP, Triple P system for foster parents.

#### Child developmental and academic outcomes

Overall, eight studies assessed child developmental outcomes in 853 children, with follow‐up between 4 months and 4 years. Seven studies (819 participants) found evidence of intervention effects on developmental outcomes, including: child‐receptive vocabulary (Bernard et al., 2017; Lewis‐Morrarty et al., 2012; Raby et al., 2019); cognitive flexibility and control (Lewis‐Morrarty et al., 2012; Lind et al., 2017); theory of mind (Lewis‐Morrarty et al., 2012); attention regulation (Lind et al., 2017); literacy (Pears et al., 2013), and school readiness (Lipscomb et al., 2013; Pears et al., 2016 found improvements in other domains such as child self‐competence and attitudes to negative behaviours for example, antisocial behaviours and alcohol use.

The quality of evidence for developmental outcomes was downgraded (−1) due to the risk of bias across the studies, giving an overall rating of ‘moderate’ quality of evidence for developmental outcomes. There were concerns in three studies regarding possible attrition bias, mainly due to a lack of information regarding loss to follow‐up and management of missing data. Concerns about the impact of lack of blinding for participants and providers were identified in two studies. At least 10 different measures were used across studies. However, there was a good level of consistency in findings, with all studies demonstrating effect sizes above 0.20, and a number of studies provided longer‐term follow‐up (up to 4 years post‐intervention). It should be noted that four of these studies measured the impact of one intervention, the ABC/ABC‐T intervention, demonstrating small effect sizes for ABC‐T and medium to large effect sizes for ABC in developmental outcome domains. The remaining two studies measured the KITS and HeadStart interventions, both of which demonstrated small effect sizes.

#### Relational outcomes

Eight studies, including 585 children, measured the effect of interventions on relational outcomes including attachment and quality of relationships. Follow‐up ranged from 0 to 12 months. Overall, five studies (464 participants), involving ABC, MTFC‐P, PCIT and HeadStart, demonstrated evidence of effects on relational outcomes, all of which demonstrated effect sizes above 0.20 in at least one domain.

Secure attachment was measured in three studies involving PCIT, ABC and MTFC‐P (Danko, 2014; Dozier et al., 2009; Fisher & Kim, 2007). None demonstrated evidence of effect sizes above 0.20 in terms of intervention‐related changes in secure attachment. Danko, 2014 reported differences in post‐intervention secure attachment when comparing two different aspects of an intervention (CDI plus PDI treatment group vs. CDI‐only control) in favour of the treatment group, although this was based on a small sample size (*n* = 34) using per protocol rather than intention to treat analyses.

Avoidant attachment was assessed in two studies of ABC and MTFC‐P (Dozier et al., 2009; Fisher & Kim, 2007), both of which reported significant reductions in avoidant behaviours at one and 12 months respectively with effect sizes above 0.20. There was no evidence for changes in resistant attachment (Fisher & Kim, 2007) or attachment disorder behaviours (Jonkman et al., 2017).

Four studies reported on the quality of relationships, including child‐caregiver and child‐teacher relationships (N'zi et al., 2016). reported improvements in child‐caregiver relationship quality and (Lipscomb et al., 2013) found evidence of positive changes in child‐teacher relationships with effect sizes above 0.20.

The quality of the evidence for relational outcomes was downgraded due to risk of bias (−1) and imprecision (−1). No studies examined relationship outcomes after 12 months; however, we did not consider that this justified additional downgrading. Three studies were rated as at high risk of bias due to lack of blinding. One study was rated as at high risk of attrition bias due to a lack of information regarding missing data. There was a large amount of heterogeneity in terms of outcome measures, with at least 10 different measures for relational outcomes used across studies.

## DISCUSSION

To our knowledge, this is the first systematic review of interventions focussing specifically on pre‐school children in foster care. Although the evidence is mixed, our findings do enable us to conclude that specific interventions are likely to be helpful in various domains for young children in foster care. In particular, there was a good level of evidence for ABC in improving developmental outcomes, including cognitive flexibility and receptive vocabulary. There appears to be some evidence for MTFC‐P, KITS, PCIT and HeadStart in reducing externalising behaviours and overall behavioural difficulties, and for ABC and MTFC‐P in reducing avoidant attachment. There was less evidence for improvements in secure attachment.

We identified 20 studies of seven different interventions. There was substantial variation in terms of outcomes and measures across studies, in keeping with previous reviews in this field (Kemmis‐Riggs et al., 2018; Kerr & Cossar, 2014; Kinsey & Schlösser, 2013). Interventions varied in terms of delivery, intensity and duration; however, most utilised either an attachment or social‐learning framework (both in some cases) (see Table 4). Within this review, just one intervention (MTFC‐P) involved birth parents as an adjunct to the intervention with foster carers. Contact with birth families has been identified by some children in foster care as being fundamental in their experience (Cleaver, 2000; Schofield & Ward, 2011) and good quality contact may promote family reunification and placement stability (Sen & Broadhurst, 2011). However, there are also concerns regarding possible harmful effects of poorly planned or poor quality contacts with birth families (Ruiz‐Romero et al., 2022; Sen & Broadhurst, 2011). It is not clear from this review whether birth‐parent involvement may have impacted child‐related outcomes and if so, in which direction.

The robustness of the findings was somewhat limited by variations in methodological quality and mixed findings across the studies, in keeping with previous reviews (Kemmis‐Riggs et al., 2018). Delivering therapeutic interventions in this context involves working with high levels of complex need within a system of uncertainty and with multiple professionals and carers involved (Kinsey & Schlösser, 2013). RCTs in social care settings frequently face challenges in terms of recruitment, retention and resource limitations (Mezey et al., 2015; Moody et al., 2021) and previous reviews have identified methodological concerns that undermine the potential for drawing robust conclusions and making firm recommendations for implementation (Dickes et al., 2018). Research within this area is notoriously challenging, given the complex nature of the difficulties faced by young people in foster care, the multidimensional and evolving nature of child protection services, and inherent variability in social care systems and provisions across different contexts. This is relevant when considering the nature of ‘treatment as usual’ (TAU) conditions, which vary depending upon local provisions and which may lead to confounding effects. For example, MTFC‐P was found to be effective in improving attachment outcomes in the Fisher & Kim, 2007 US trial but findings were unexpectedly not replicated in the Jonkman et al., 2017 Dutch study. As highlighted by study authors, this may be partly related to a difference in TAU conditions which in the Netherlands might include various evidence‐based interventions such as PCIT and Trauma Focused‐CBT.

Complementary economic evaluation studies show promising results in terms of cost‐effectiveness of MTFC‐P and KITS, providing evidence to support investment in these programs. MTFC has been demonstrated to be significantly cheaper than usual foster care, leading to a positive net benefit in terms of incremental permanent placement for a range of willingness to pay thresholds (Lynch et al., 2014). KITS has been also found to be cost‐effective, leading to increased days free from internalising symptoms and externalizing behaviours at a relatively modest cost (Lynch et al., 2017).

We did not identify any existing RCTs of several interventions that have been identified in previous studies as being routinely used in the UK for children with or at‐risk of attachment difficulties, including Individual Child Psychotherapy (ICP), Dyadic Developmental Psychotherapy (DDP), Theraplay, Circle of Security (COS) and Watch, Wait and Wonder (WWW) (Wright et al., 2023), suggesting that further research is needed in determining whether other interventions may be helpful.

Due to heterogeneity between studies, it was difficult to draw firm conclusions regarding subgroup differences and possible impacts on interventions. For example, factors such as maltreatment history, caregiver type, child age and number of foster placements might influence the differential effectiveness of these interventions. Few studies included mediator and moderator analyses, likely due to limitations related to small sample sizes and power. These analyses would be helpful in understanding which interventions work for whom in which context (MacKinnon, 2011), which is particularly important for looked after children given the complexity of their experiences. For example, effects of ABC may be moderated by child age: ABC appears to improve behavioural outcomes in toddlers aged 18–36 months but not in infants aged 0–17 months (Dozier et al., 2006). In contrast, children enroled in foster care at a younger age experienced greater increases in secure attachment behaviours over time (Fisher & Kim, 2007), replicating findings from previous research (Stovall‐McClough & Dozier 2004).

The range of outcomes in this review were broad, reflecting that psychological wellbeing in young children is multi‐dimensional. Parent‐related outcomes are considered in a separate paper (in process). This may be considered a limitation of the current review, given what we know about the important relationships between child and caregiver outcomes—for example, the associations between maternal sensitivity and attachment security (Bakermans‐Kranenburg et al., 2003). However, there are strong arguments for a focus solely on child‐related outcomes in order to emphasise the voice of the child, which can be often be lost within the context of large and complex systems such as social care (McFadyen et al., 2023). Our review included RCTs only which limits the breadth of our findings, particularly in terms of understanding longer‐term outcomes in this group. This may be particularly important in understanding attachment‐related outcomes whereby studies indicate that moving from insecure to secure attachment styles can be difficult within short time‐frames, particularly for children who enter foster care after 12 months of age, even where environmental conditions improve (Rushton et al., 2003). This may provide some explanation as to the limited evidence for changes in secure attachment demonstrated in this review, as follow‐up was limited to under 12 months for these particular studies. Other limitations include the lack of full independent screening for title/abstracts, although this is somewhat mitigated by high inter‐rater reliability scores on a subsample of records, and lack of blinding for data extraction. Some efforts were made to identify grey literature (e.g. references searches, Internet engine searches) and we did identify a number of unpublished papers (including one paper, Danko, 2014, included in the final review). However, comprehensive searches of grey literature were not undertaken which may have limited the scope of our findings.

## CONCLUSION

In summary, this systematic review was able to support our understanding of which interventions may be helpful in improving outcomes for pre‐school children in foster care. The growing field of research in this area is promising and researchers continue to strive to develop our understanding of this complex area, despite the challenging nature of work in this field. However, there continue to be significant gaps in the research evidence, particularly in achieving the methodological rigour necessary to ensure confidence in findings and in understanding how important moderators might impact upon the effectiveness of interventions. In an effort to detract from the problem‐saturated narrative that often surrounds work in this field, studies that enable us to better understand the strengths and resilience of children and carers would be welcomed. Heterogeneity across studies creates difficulty in synthesizing results (Dickes et al., 2018) and in making recommendations as to how and when specific interventions might be best implemented. Despite these limitations, our findings do suggest that some interventions are likely to be helpful in this group of children. Previous research has identified poor availability of evidence‐based interventions in UK Health and Social Care, highlighting the need for dissemination of evidence based approaches (Wright et al., 2023). Apart from ABC, none of the interventions included within this review have been identified as being routinely used in UK Health and Social Care (Wright et al., 2023). We suggest that interventions with evidence of effectiveness (ABC, MTFC‐P, KITS, HeadStart and PCIT) should begin to be implemented and evaluated within clinical practice. Building on what we know, future research should focus on evaluating the effects of relevant interventions with coherent and consistent use of appropriate outcome measures across studies, in order to support a more robust understanding of what works well for young children in foster care.

## AUTHOR CONTRIBUTION

**Natalie Kirby**: Conceptualization; formal analysis; methodology; project administration; writing – original draft; writing – review & editing. **Camilla Biggs**: Conceptualization; formal analysis; methodology; writing – original draft; writing – review & editing. **Megan Garside**: Formal analysis; methodology; writing – original draft; writing – review & editing. **Gloria Cheung**: Formal analysis; methodology; writing – original draft. **Philip Wilson**: Writing – review & editing. **Matt Forde**: Writing – review & editing. **Manuela Deidda**: Writing – review & editing. **Dennis Ougrin**: Writing – review & editing. **Fiona Turner**: Writing – review & editing. **Karen Crawford**: Writing – review & editing. **Helen Minnis**: Conceptualization; methodology; project administration; supervision; writing – review & editing.

## CONFLICT OF INTEREST STATEMENT

The authors declare no conflicts of interest.

## ETHICAL CONSIDERATIONS

The Best Services Trial (BeST?) was approved by the West of Scotland Research Ethics Committee.

## Supporting information

Supporting Information S1

Supporting Information S2

## Data Availability

Data sharing not applicable to this article as no datasets were generated or analysed during the current study.
