# Supplementary material for: Interventions for pre‐school children in foster care: A systematic review of randomised controlled trials of child‐related outcomes
Source: JCPP Adv. 2024 Sep 5;5(1):e12273. doi: 10.1002/jcv2.12273 (PMC11889642; doi:10.1002/jcv2.12273)
Supplement: Supplementary file 1 — Supporting Information S1 [file JCV2-5-e12273-s001.docx]

**Supplementary information**

**Search strategy**

Embase: search run on 23/06/2023

Embase 1947-Present, updated daily

1 Randomized Controlled Trial/ 792970

2 controlled clinical trial/ 469842

3 multicenter study/ 381029

4 Phase 3 clinical trial/ 70162

5 Phase 4 clinical trial/ 5491

6 exp RANDOMIZATION/ 100085

7 Single Blind Procedure/ 52285

8 Double Blind Procedure/ 213997

9 Crossover Procedure/ 75909

10 PLACEBO/ 415359

11 randomi?ed controlled trial$.tw. 329121

12 rct.tw. 54261

13 (random$ adj2 allocat$).tw. 55255

14 single blind$.tw. 32071

15 double blind$.tw. 251679

16 ((treble or triple) adj blind$).tw. 1944

17 placebo$.tw. 375098

18 Prospective Study/ 886681

19 Clinical Trial/ 1099294

20 or/1-19 2997179

21 Case Study/ 108721

22 case report.tw. 563927

23 abstract report/ or letter/ 1322362

24 Conference proceedings.pt. 0

25 conference abstract.pt. 4803490

26 Editorial.pt. 781034

27 Letter.pt. 1309953

28 Note.pt. 945649

29 or/21-28 8430819

30 20 not 29 2178212

31 babies.ti,ab,kf. 67852

32 baby.ti,ab,kf. 69620

33 infan*.ti,ab,kf. 687229

34 neonat*.ti,ab,kf. 440023

35 neo-nat*.ti,ab,kf. 634

36 newborn*.ti,ab,kf. 265108

37 new-born*.ti,ab,kf. 9388

38 perinat*.ti,ab,kf. 130911

39 hospitalized infant/ 1047

40 infant care/ 1755

41 high risk infant/ 3839

42 infant/ 812061

43 small for date infant/ 19707

44 prematurity/ 141518

45 premature.ti,ab,kf. 205955

46 large for gestational age/ 4830

47 newborn/ 695122

48 boy?.ti,ab,kf. 245032

49 boyfrien*.ti,ab,kf. 964

50 boyhood*.ti,ab,kf. 117

51 child*.ti,ab,kf. 2310733

52 child/ 2379140

53 child care/ 42305

54 preschool child/ 699691

55 school child/ 433266

56 fifth-grader*.ti,ab,kf. 556

57 first-grader*.ti,ab,kf. 671

58 fourth-grader*.ti,ab,kf. 472

59 girl/ 58970

60 girl?.ti,ab,kf. 252770

61 boy/ 46903

62 girlfrien*.ti,ab,kf. 693

63 girlhood*.ti,ab,kf. 44

64 juvenil*.ti,ab,kf. 126368

65 kid?.ti,ab,kf. 16923

66 kindergarten*.ti,ab,kf. 9812

67 minor?.ti,ab,kf. 354013

68 "minor (person)"/ 908

69 minority.ti,ab,kf. 110967

70 paediatric*.ti,ab,kf. 152085

71 pediatrics/ 102712

72 pediatric*.ti,ab,kf. 623331

73 pediatrics/ 102712

74 PICU.ti,ab,kf. 15719

75 preschool*.ti,ab,kf. 44464

76 preschool child/ 699691

77 pre-school*.ti,ab,kf. 8823

78 second-grader*.ti,ab,kf. 428

79 seventh-grader*.ti,ab,kf. 368

80 sixth-grader*.ti,ab,kf. 621

81 stepchild*.ti,ab,kf. 361

82 step-child*.ti,ab,kf. 81

83 stepchild/ 45

84 third-grader*.ti,ab,kf. 406

85 young*.ti,ab,kf. 1163953

86 toddler?.ti,ab,kf. 18699

87 (young adj2 person).ti,ab,kf. 2557

88 youngster.tw. 369

89 youth*.tw. 123320

90 juvenile/ 64857

91 nursery.ti,ab,kf. 12970

92 primary school.ti,ab,kf. 15508

93 reception.ti,ab,kf. 13500

94 primary school/ 17546

95 nursery/ 5649

96 nursery school/ 1774

97 or/31-96 6089373

98 exp Foster Home Care/ 5689

99 Foster care/ 5689

100 foster-care$.tw. 3218

101 (foster adj3 care$).tw. 4181

102 (foster adj3 parent$).tw. 975

103 (foster adj3 mother$).tw. 794

104 (foster adj3 father$).tw. 49

105 (foster adj3 child$).tw. 1839

106 ((kin or kinship or kindred) adj3 care$).tw. 561

107 ((kin or kinship or kindred) adj3 parent$).tw. 253

108 ((kin or kinship or kindred) adj3 mother$).tw. 79

109 ((kin or kinship or kindred) adj3 father$).tw. 31

110 ((kinship or kindred) adj3 child$).tw. 153

111 "living in care".tw. 402

112 "legal guardian".tw. 531

113 guardian*.tw. 14914

114 ("local authority" adj3 care$).tw. 150

115 (child adj3 placement).tw. 367

116 ("out of home" adj3 placement).tw. 361

117 ("out of home" adj3 care$).tw. 839

118 (alternat* adj3 care$).tw. 6289

119 ((relation or relative) adj3 care$).tw. 6537

120 or/98-119 38198

121 30 and 97 and 120 1896

Medline 23062023

Ovid MEDLINE(R) ALL <1946 to June 22, 2023>

1 Randomized Controlled Trials as Topic/ 162627

2 randomized controlled trial/ 595054

3 Random Allocation/ 106940

4 Double Blind Method/ 175500

5 Single Blind Method/ 32771

6 clinical trial/ 538195

7 clinical trial,phase i.pt. 24947

8 clinical trail, phase ii.pt. 0

9 clinical trial, phase iii.pt. 21784

10 clinical trial, phase iv.pt. 2419

11 controlled clinical trial.pt. 95340

12 randomized controlled trial.pt. 595054

13 multicenter study.pt. 335045

14 clinical trial.pt. 538195

15 exp Clinical Trials as topic/ 382851

16 or/1-15 1558509

17 (clinical adj trial$).tw. 477378

18 ((singl$ or double$ or trb$ or tripl$) adj (blind$3 or mask$3)).tw. 197474

19 PLACEBOS/ 35930

20 placebo$.tw. 246931

21 randomly allocated.tw. 36309

22 (allocated adj2 random$).tw. 40071

23 or/17-22 781328

24 16 or 23 1905416

25 case report.tw. 396191

26 letter/ 1220598

27 historical article/ 369327

28 or/25-27 1967134

29 24 not 28 1863014

30 exp Foster Home Care/ 3890

31 Foster care/ 3890

32 Child, Foster/ 191

33 foster-care$.tw. 2765

34 (foster adj3 care$).tw. 3514

35 (foster adj3 parent$).tw. 825

36 (foster adj3 mother$).tw. 616

37 (foster adj3 father$).tw. 51

38 (foster adj3 child$).tw. 1584

39 ((kin or kinship or kindred) adj3 care$).tw. 508

40 ((kin or kinship or kindred) adj3 parent$).tw. 219

41 ((kinship or kindred) adj3 mother$).tw. 37

42 ((kin or kinship or kindred) adj3 father$).tw. 28

43 ((kin or kinship or kindred) adj3 child$).tw. 231

44 "living in care".tw. 280

45 guardian*.tw. 10160

46 ("local authority" adj3 care$).tw. 113

47 (child adj3 placement).tw. 303

48 ("out of home" adj3 placement).tw. 332

49 ("out of home" adj3 care$).tw. 758

50 (alternat* adj3 care$).tw. 4688

51 ((relation or relative) adj3 care$).tw. 4978

52 or/30-51 28245

53 babies.ti,ab,kf. 41963

54 baby.ti,ab,kf. 44677

55 infan*.ti,ab,kf. 551213

56 neonat*.ti,ab,kf. 313486

57 neo-nat*.ti,ab,kf. 313

58 newborn*.ti,ab,kf. 197449

59 new-born*.ti,ab,kf. 5023

60 perinat*.ti,ab,kf. 91320

61 premature.ti,ab,kf. 141374

62 Infant, Extremely Premature/ 3866

63 Infant, Large for Gestational Age/ 4

64 Infant, Newborn/ 668485

65 Infant, Postmature/ 396

66 Infant, Premature/ 61379

67 Infant, Small for Gestational Age/ 8597

68 Infant/ 865370

69 boy?.ti,ab,kf. 166259

70 boyfrien*.ti,ab,kf. 747

71 boyhood*.ti,ab,kf. 92

72 child*.ti,ab,kf. 1672934

73 fifth-grader*.ti,ab,kf. 546

74 first-grader*.ti,ab,kf. 629

75 fourth-grader*.ti,ab,kf. 463

76 girl?.ti,ab,kf. 171616

77 girlfrien*.ti,ab,kf. 527

78 girlhood*.ti,ab,kf. 42

79 juvenil*.ti,ab,kf. 95893

80 kid?.ti,ab,kf. 10899

81 kindergarten*.ti,ab,kf. 8178

82 minor?.ti,ab,kf. 258733

83 minority.ti,ab,kf. 79514

84 paediatric*.ti,ab,kf. 85594

85 pediatric*.ti,ab,kf. 387461

86 PICU.ti,ab,kf. 6747

87 preschool*.ti,ab,kf. 35604

88 pre-school*.ti,ab,kf. 5880

89 second-grader*.ti,ab,kf. 402

90 seventh-grader*.ti,ab,kf. 356

91 sixth-grader*.ti,ab,kf. 593

92 stepchild*.ti,ab,kf. 291

93 step-child*.ti,ab,kf. 54

94 third-grader*.ti,ab,kf. 412

95 toddler?.ti,ab,kf. 13949

96 young*.ti,ab,kf. 819542

97 youngster.tw. 201

98 youth*.tw. 96308

99 Child/ 1910885

100 Child, Preschool/ 989207

101 (young adj2 person).ti,ab,kf. 1418

102 nursery.ti,ab,kf. 10660

103 primary school.ti,ab,kf. 11619

104 reception.ti,ab,kf. 10481

105 primary school/ 51241

106 nursery school/ 1506

107 girl/ 15181

108 boy/ 3953

109 or/53-108 4699229

110 29 and 52 and 109 1394

CInahl 23062023

*this search included a number of ‘legal notes’ from the journal Adoption and Fostering which were removed*

|  | | | | |
| --- | --- | --- | --- | --- |
| **#** | **Query** | **Limiters/Expanders** | **Last Run Via** | **Results** |
| S45 | S10 AND S32 AND S44 | Expanders - Apply equivalent subjects  Search modes - Boolean/Phrase | Interface - EBSCOhost Research Databases  Search Screen - Advanced Search  Database - CINAHL | 3,683 |
| S44 | S33 or S34 or S35 or S36 or S37 or S38 or S39 or S40 or S41 or S42 or S43 | Expanders - Apply equivalent subjects  Search modes - Boolean/Phrase | Interface - EBSCOhost Research Databases  Search Screen - Advanced Search  Database - CINAHL | 1,706,859 |
| S43 | TX allocat* random* | Expanders - Apply equivalent subjects  Search modes - Boolean/Phrase | Interface - EBSCOhost Research Databases  Search Screen - Advanced Search  Database - CINAHL | 16,090 |
| S42 | (MH "Quantitative Studies") | Expanders - Apply equivalent subjects  Search modes - Boolean/Phrase | Interface - EBSCOhost Research Databases  Search Screen - Advanced Search  Database - CINAHL | 35,635 |
| S41 | (MH "Placebos") | Expanders - Apply equivalent subjects  Search modes - Boolean/Phrase | Interface - EBSCOhost Research Databases  Search Screen - Advanced Search  Database - CINAHL | 13,711 |
| S40 | TX placebo* | Expanders - Apply equivalent subjects  Search modes - Boolean/Phrase | Interface - EBSCOhost Research Databases  Search Screen - Advanced Search  Database - CINAHL | 79,431 |
| S39 | TX random* allocat* | Expanders - Apply equivalent subjects  Search modes - Boolean/Phrase | Interface - EBSCOhost Research Databases  Search Screen - Advanced Search  Database - CINAHL | 16,090 |
| S38 | (MH "Random Assignment") | Expanders - Apply equivalent subjects  Search modes - Boolean/Phrase | Interface - EBSCOhost Research Databases  Search Screen - Advanced Search  Database - CINAHL | 79,552 |
| S37 | TX randomi* control* trial* | Expanders - Apply equivalent subjects  Search modes - Boolean/Phrase | Interface - EBSCOhost Research Databases  Search Screen - Advanced Search  Database - CINAHL | 261,901 |
| S36 | TX ( (singl* n1 blind*) or (singl* n1 mask*) ) or TX ( (doubl* n1 blind*) or (doubl* n1 mask*) ) or TX ( (tripl* n1 blind*) or (tripl* n1 mask*) ) or TX ( (trebl* n1 blind*) or (trebl* n1 mask*) ) | Expanders - Apply equivalent subjects  Search modes - Boolean/Phrase | Interface - EBSCOhost Research Databases  Search Screen - Advanced Search  Database - CINAHL | 1,262,393 |
| S35 | TX clinic* n1 trial* | Expanders - Apply equivalent subjects  Search modes - Boolean/Phrase | Interface - EBSCOhost Research Databases  Search Screen - Advanced Search  Database - CINAHL | 335,937 |
| S34 | PT Clinical trial | Expanders - Apply equivalent subjects  Search modes - Boolean/Phrase | Interface - EBSCOhost Research Databases  Search Screen - Advanced Search  Database - CINAHL | 113,337 |
| S33 | MH "Clinical Trials+" | Expanders - Apply equivalent subjects  Search modes - Boolean/Phrase | Interface - EBSCOhost Research Databases  Search Screen - Advanced Search  Database - CINAHL | 348,682 |
| S32 | S11 OR S12 OR S13 OR S14 OR S15 OR S16 OR S17 OR S18 OR S19 OR S20 OR S21 OR S22 OR S23 OR S24 OR S25 OR S26 OR S27 OR S28 OR S29 OR S30 OR S31 | Expanders - Apply equivalent subjects  Search modes - Boolean/Phrase | Interface - EBSCOhost Research Databases  Search Screen - Advanced Search  Database - CINAHL | 23,071 |
| S31 | TI (relation or relative) N3 care$ OR AB (relation or relative) N3 care$ | Expanders - Apply equivalent subjects  Search modes - Boolean/Phrase | Interface - EBSCOhost Research Databases  Search Screen - Advanced Search  Database - CINAHL | 5,415 |
| S30 | TI alternat* N3 care$ OR AB alternat* N3 care$ | Expanders - Apply equivalent subjects  Search modes - Boolean/Phrase | Interface - EBSCOhost Research Databases  Search Screen - Advanced Search  Database - CINAHL | 3,480 |
| S29 | TI "out of home" N3 care OR AB "out of home" N3 care | Expanders - Apply equivalent subjects  Search modes - Boolean/Phrase | Interface - EBSCOhost Research Databases  Search Screen - Advanced Search  Database - CINAHL | 890 |
| S28 | TI "out of home" N3 placement OR AB "out of home" N3 placement | Expanders - Apply equivalent subjects  Search modes - Boolean/Phrase | Interface - EBSCOhost Research Databases  Search Screen - Advanced Search  Database - CINAHL | 394 |
| S27 | TI "child placement" OR AB local authority N3 care$ | Expanders - Apply equivalent subjects  Search modes - Boolean/Phrase | Interface - EBSCOhost Research Databases  Search Screen - Advanced Search  Database - CINAHL | 287 |
| S26 | TI local authority N3 care$ OR AB local authority N3 care$ | Expanders - Apply equivalent subjects  Search modes - Boolean/Phrase | Interface - EBSCOhost Research Databases  Search Screen - Advanced Search  Database - CINAHL | 284 |
| S25 | TI guardian* OR AB guardian* | Expanders - Apply equivalent subjects  Search modes - Boolean/Phrase | Interface - EBSCOhost Research Databases  Search Screen - Advanced Search  Database - CINAHL | 4,225 |
| S24 | TI "living in care" OR AB "living in care" | Expanders - Apply equivalent subjects  Search modes - Boolean/Phrase | Interface - EBSCOhost Research Databases  Search Screen - Advanced Search  Database - CINAHL | 295 |
| S23 | TI ( (kin or kinship or kindred) N3 child$ ) OR AB ( (kin or kinship or kindred) N3 child$ ) | Expanders - Apply equivalent subjects  Search modes - Boolean/Phrase | Interface - EBSCOhost Research Databases  Search Screen - Advanced Search  Database - CINAHL | 286 |
| S22 | TI ( (kin or kinship or kindred) N3 father$ ) OR AB ( (kin or kinship or kindred) N3 father$ ) | Expanders - Apply equivalent subjects  Search modes - Boolean/Phrase | Interface - EBSCOhost Research Databases  Search Screen - Advanced Search  Database - CINAHL | 15 |
| S21 | TI ( (kin or kinship or kindred) N3 mother$ ) OR AB ( (kin or kinship or kindred) N3 mother$ ) | Expanders - Apply equivalent subjects  Search modes - Boolean/Phrase | Interface - EBSCOhost Research Databases  Search Screen - Advanced Search  Database - CINAHL | 27 |
| S20 | TI ( (kin or kinship or kindred) N3 parent$ ) OR AB ( (kin or kinship or kindred) N3 parent$ ) | Expanders - Apply equivalent subjects  Search modes - Boolean/Phrase | Interface - EBSCOhost Research Databases  Search Screen - Advanced Search  Database - CINAHL | 89 |
| S19 | TI ( (kin or kinship or kindred) N3 care$ ) OR AB ( (kin or kinship or kindred) N3 care$ ) | Expanders - Apply equivalent subjects  Search modes - Boolean/Phrase | Interface - EBSCOhost Research Databases  Search Screen - Advanced Search  Database - CINAHL | 562 |
| S18 | TI foster N3 child$ OR AB foster N3 child$ | Expanders - Apply equivalent subjects  Search modes - Boolean/Phrase | Interface - EBSCOhost Research Databases  Search Screen - Advanced Search  Database - CINAHL | 1,935 |
| S17 | TI foster N3 father$ OR AB foster N3 father$ | Expanders - Apply equivalent subjects  Search modes - Boolean/Phrase | Interface - EBSCOhost Research Databases  Search Screen - Advanced Search  Database - CINAHL | 39 |
| S16 | TI foster N3 mother$ OR AB foster N3 mother$ | Expanders - Apply equivalent subjects  Search modes - Boolean/Phrase | Interface - EBSCOhost Research Databases  Search Screen - Advanced Search  Database - CINAHL | 148 |
| S15 | TI foster N3 parent$ OR AB foster N3 parent$ | Expanders - Apply equivalent subjects  Search modes - Boolean/Phrase | Interface - EBSCOhost Research Databases  Search Screen - Advanced Search  Database - CINAHL | 888 |
| S14 | TI foster N3 care$ OR AB foster N3 care$ | Expanders - Apply equivalent subjects  Search modes - Boolean/Phrase | Interface - EBSCOhost Research Databases  Search Screen - Advanced Search  Database - CINAHL | 4,048 |
| S13 | TI foster care OR AB foster care | Expanders - Apply equivalent subjects  Search modes - Boolean/Phrase | Interface - EBSCOhost Research Databases  Search Screen - Advanced Search  Database - CINAHL | 4,450 |
| S12 | TI foster-care* OR AB foster-care* | Expanders - Apply equivalent subjects  Search modes - Boolean/Phrase | Interface - EBSCOhost Research Databases  Search Screen - Advanced Search  Database - CINAHL | 3,567 |
| S11 | (MH "Foster Home Care") OR (MH "Foster Parents") OR (MH "Child, Foster") | Expanders - Apply equivalent subjects  Search modes - Boolean/Phrase | Interface - EBSCOhost Research Databases  Search Screen - Advanced Search  Database - CINAHL | 6,777 |
| S10 | S1 OR S2 OR S3 OR S4 OR S5 OR S6 OR S7 OR S8 OR S9 | Expanders - Apply equivalent subjects  Search modes - Boolean/Phrase | Interface - EBSCOhost Research Databases  Search Screen - Advanced Search  Database - CINAHL | 1,280,771 |
| S9 | (MH "Schools, Nursery") | Expanders - Apply equivalent subjects  Search modes - Boolean/Phrase | Interface - EBSCOhost Research Databases  Search Screen - Advanced Search  Database - CINAHL | 1,268 |
| S8 | MH(young person) | Expanders - Apply equivalent subjects  Search modes - SmartText Searching | Interface - EBSCOhost Research Databases  Search Screen - Advanced Search  Database - CINAHL | 1,372 |
| S7 | TI (young N2 (person)) OR AB ( young N2 (person)) | Expanders - Apply equivalent subjects  Search modes - Boolean/Phrase | Interface - EBSCOhost Research Databases  Search Screen - Advanced Search  Database - CINAHL | 2,149 |
| S6 | MH ("Child" OR "Child, Preschool") | Expanders - Apply equivalent subjects  Search modes - Boolean/Phrase | Interface - EBSCOhost Research Databases  Search Screen - Advanced Search  Database - CINAHL | 585,018 |
| S5 | AB (boy# OR boyfrien* OR boyhood* OR child* OR fifth-grader* OR first-grader* OR fourth-grader* OR girl# OR girlfriend* OR girlhood* OR juvenil * OR kid# OR kindergarten* OR minor# OR minority OR paediatric* OR peadiatric* OR pediatric* OR PICU OR preschool* OR pre-school* OR second-grader* OR seventh-grader* OR sixth-grader* OR stepchild* OR step-child* OR third-grader* OR toddler# OR young OR youngster* OR youth*) | Expanders - Apply equivalent subjects  Search modes - Boolean/Phrase | Interface - EBSCOhost Research Databases  Search Screen - Advanced Search  Database - CINAHL | 706,321 |
| S4 | TI (boy# OR boyfrien* OR boyhood* OR child* OR fifth-grader* OR first-grader* OR fourth-grader* OR girl# OR girlfriend* OR girlhood* OR juvenil* OR kid# OR kindergarten* OR minor# OR minority OR paediatric* OR peadiatric* OR pediatric* OR PICU OR preschool* OR pre-school* OR second-grader* OR seventh-grader* OR sixth-grader* OR stepchild* OR step-child* OR third-grader* OR toddler # OR young OR youngster* OR youth*) | Expanders - Apply equivalent subjects  Search modes - Boolean/Phrase | Interface - EBSCOhost Research Databases  Search Screen - Advanced Search  Database - CINAHL | 550,796 |
| S3 | MH ("Infant, Newborn" OR "Infant") | Expanders - Apply equivalent subjects  Search modes - Boolean/Phrase | Interface - EBSCOhost Research Databases  Search Screen - Advanced Search  Database - CINAHL | 275,408 |
| S2 | AB (babies OR baby OR infan* OR neonat* OR neo-nat* OR newborn* OR new -born* OR perinat*) | Expanders - Apply equivalent subjects  Search modes - Boolean/Phrase | Interface - EBSCOhost Research Databases  Search Screen - Advanced Search  Database - CINAHL | 182,674 |
| S1 | TI (babies OR baby OR infan* OR neonat* OR neo-nat* OR newborn* OR new-born* OR perinat*) | Expanders - Apply equivalent subjects  Search modes - Boolean/Phrase | Interface - EBSCOhost Research Databases  Search Screen - Advanced Search  Database - CINAHL | 137,715 |

PsycInfo

| **Query** | **Limiters/Expanders** | **Last Run Via** | **Results** |  |
| --- | --- | --- | --- | --- |
| S91 | S56 AND S78 AND S90 | Expanders - Apply equivalent subjects Search modes - Boolean/Phrase | Interface - EBSCOhost Research Databases Search Screen - Advanced Search Database - APA PsycInfo | 449 |
| S90 | S79 or S80 or S81 or S82 or S83 or S84 or S85 or S86 or S87 or S88 or S89 | Expanders - Apply equivalent subjects Search modes - Boolean/Phrase | Interface - EBSCOhost Research Databases Search Screen - Advanced Search Database - APA PsycInfo | 167,811 |
| S89 | TX allocat* random* | Expanders - Apply equivalent subjects Search modes - Boolean/Phrase | Interface - EBSCOhost Research Databases Search Screen - Advanced Search Database - APA PsycInfo | 13,743 |
| S88 | (DE "Quantitative Methods") | Expanders - Apply equivalent subjects Search modes - Boolean/Phrase | Interface - EBSCOhost Research Databases Search Screen - Advanced Search Database - APA PsycInfo | 3,963 |
| S87 | (DE "Placebo") | Expanders - Apply equivalent subjects Search modes - Boolean/Phrase | Interface - EBSCOhost Research Databases Search Screen - Advanced Search Database - APA PsycInfo | 6,499 |
| S86 | TX placebo* | Expanders - Apply equivalent subjects Search modes - Boolean/Phrase | Interface - EBSCOhost Research Databases Search Screen - Advanced Search Database - APA PsycInfo | 45,493 |
| S85 | TX random* allocat* | Expanders - Apply equivalent subjects Search modes - Boolean/Phrase | Interface - EBSCOhost Research Databases Search Screen - Advanced Search Database - APA PsycInfo | 13,743 |
| S84 | DE "Random Sampling" | Expanders - Apply equivalent subjects Search modes - Boolean/Phrase | Interface - EBSCOhost Research Databases Search Screen - Advanced Search Database - APA PsycInfo | 955 |
| S83 | TX randomi* control* trial* | Expanders - Apply equivalent subjects Search modes - Boolean/Phrase | Interface - EBSCOhost Research Databases Search Screen - Advanced Search Database - APA PsycInfo | 59,247 |
| S82 | TX ( (singl* n1 blind*) or (singl* n1 mask*) ) or TX ( (doubl* n1 blind*) or (doubl* n1 mask*) ) or TX ( (tripl* n1 blind*) or (tripl* n1 mask*) ) or TX ( (trebl* n1 blind*) or (trebl* n1 mask*) ) | Expanders - Apply equivalent subjects Search modes - Boolean/Phrase | Interface - EBSCOhost Research Databases Search Screen - Advanced Search Database - APA PsycInfo | 37,025 |
| S81 | TX clinic* n1 trial* | Expanders - Apply equivalent subjects Search modes - Boolean/Phrase | Interface - EBSCOhost Research Databases Search Screen - Advanced Search Database - APA PsycInfo | 84,415 |
| S80 | PT Clinical trial | Expanders - Apply equivalent subjects Search modes - Boolean/Phrase | Interface - EBSCOhost Research Databases Search Screen - Advanced Search Database - APA PsycInfo | 1,042 |
| S79 | DE "Clinical Trials+" OR DE "Randomized Controlled Trials" OR DE "Randomized Clinical Trials" | Expanders - Apply equivalent subjects Search modes - Boolean/Phrase | Interface - EBSCOhost Research Databases Search Screen - Advanced Search Database - APA PsycInfo | 1,455 |
| S78 | S57 OR S58 OR S59 OR S60 OR S61 OR S62 OR S63 OR S64 OR S65 OR S66 OR S67 OR S68 OR S69 OR S70 OR S71 OR S72 OR S73 OR S74 OR S75 OR S76 OR S77 | Expanders - Apply equivalent subjects Search modes - Boolean/Phrase | Interface - EBSCOhost Research Databases Search Screen - Advanced Search Database - APA PsycInfo | 25,272 |
| S77 | TI (relation or relative) N3 care$ OR AB (relation or relative) N3 care$ | Expanders - Apply equivalent subjects Search modes - Boolean/Phrase | Interface - EBSCOhost Research Databases Search Screen - Advanced Search Database - APA PsycInfo | 3,720 |
| S76 | TI alternat* N3 care$ OR AB alternat* N3 care$ | Expanders - Apply equivalent subjects Search modes - Boolean/Phrase | Interface - EBSCOhost Research Databases Search Screen - Advanced Search Database - APA PsycInfo | 1,929 |
| S75 | TI "out of home" N3 care OR AB "out of home" N3 care | Expanders - Apply equivalent subjects Search modes - Boolean/Phrase | Interface - EBSCOhost Research Databases Search Screen - Advanced Search Database - APA PsycInfo | 1,447 |
| S74 | TI "out of home" N3 placement OR AB "out of home" N3 placement | Expanders - Apply equivalent subjects Search modes - Boolean/Phrase | Interface - EBSCOhost Research Databases Search Screen - Advanced Search Database - APA PsycInfo | 1,104 |
| S73 | TI "child placement" OR AB local authority N3 care$ | Expanders - Apply equivalent subjects Search modes - Boolean/Phrase | Interface - EBSCOhost Research Databases Search Screen - Advanced Search Database - APA PsycInfo | 279 |
| S72 | TI local authority N3 care$ OR AB local authority N3 care$ | Expanders - Apply equivalent subjects Search modes - Boolean/Phrase | Interface - EBSCOhost Research Databases Search Screen - Advanced Search Database - APA PsycInfo | 221 |
| S71 | TI guardian* OR AB guardian | Expanders - Apply equivalent subjects Search modes - Boolean/Phrase | Interface - EBSCOhost Research Databases Search Screen - Advanced Search Database - APA PsycInfo | 4,523 |
| S70 | TI "living in care" OR AB "living in care" | Expanders - Apply equivalent subjects Search modes - Boolean/Phrase | Interface - EBSCOhost Research Databases Search Screen - Advanced Search Database - APA PsycInfo | 300 |
| S69 | TI ( (kin or kinship or kindred) N3 child$ ) OR AB ( (kin or kinship or kindred) N3 child$ ) | Expanders - Apply equivalent subjects Search modes - Boolean/Phrase | Interface - EBSCOhost Research Databases Search Screen - Advanced Search Database - APA PsycInfo | 610 |
| S68 | TI ( (kin or kinship or kindred) N3 father$ ) OR AB ( (kin or kinship or kindred) N3 father$ ) | Expanders - Apply equivalent subjects Search modes - Boolean/Phrase | Interface - EBSCOhost Research Databases Search Screen - Advanced Search Database - APA PsycInfo | 64 |
| S67 | TI ( (kin or kinship or kindred) N3 mother$ ) OR AB ( (kin or kinship or kindred) N3 mother$ ) | Expanders - Apply equivalent subjects Search modes - Boolean/Phrase | Interface - EBSCOhost Research Databases Search Screen - Advanced Search Database - APA PsycInfo | 137 |
| S66 | TI ( (kin or kinship or kindred) N3 parent$ ) OR AB ( (kin or kinship or kindred) N3 parent$ ) | Expanders - Apply equivalent subjects Search modes - Boolean/Phrase | Interface - EBSCOhost Research Databases Search Screen - Advanced Search Database - APA PsycInfo | 275 |
| S65 | TI ( (kin or kinship or kindred) N3 care$ ) OR AB ( (kin or kinship or kindred) N3 care$ ) | Expanders - Apply equivalent subjects Search modes - Boolean/Phrase | Interface - EBSCOhost Research Databases Search Screen - Advanced Search Database - APA PsycInfo | 858 |
| S64 | TI foster N3 child$ OR AB foster N3 child$ | Expanders - Apply equivalent subjects Search modes - Boolean/Phrase | Interface - EBSCOhost Research Databases Search Screen - Advanced Search Database - APA PsycInfo | 4,841 |
| S63 | TI foster N3 father$ OR AB foster N3 father$ | Expanders - Apply equivalent subjects Search modes - Boolean/Phrase | Interface - EBSCOhost Research Databases Search Screen - Advanced Search Database - APA PsycInfo | 152 |
| S62 | TI foster N3 mother$ OR AB foster N3 mother$ | Expanders - Apply equivalent subjects Search modes - Boolean/Phrase | Interface - EBSCOhost Research Databases Search Screen - Advanced Search Database - APA PsycInfo | 563 |
| S61 | TI foster N3 parent$ OR AB foster N3 parent$ | Expanders - Apply equivalent subjects Search modes - Boolean/Phrase | Interface - EBSCOhost Research Databases Search Screen - Advanced Search Database - APA PsycInfo | 2,603 |
| S60 | TI foster N3 care$ OR AB foster N3 care$ | Expanders - Apply equivalent subjects Search modes - Boolean/Phrase | Interface - EBSCOhost Research Databases Search Screen - Advanced Search Database - APA PsycInfo | 7,364 |
| S59 | TI foster care OR AB foster care | Expanders - Apply equivalent subjects Search modes - Boolean/Phrase | Interface - EBSCOhost Research Databases Search Screen - Advanced Search Database - APA PsycInfo | 7,678 |
| S58 | TI foster-care* OR AB foster-care* | Expanders - Apply equivalent subjects Search modes - Boolean/Phrase | Interface - EBSCOhost Research Databases Search Screen - Advanced Search Database - APA PsycInfo | 6,916 |
| S57 | DE "Foster Care" OR DE "Foster Parents" OR DE "Foster Children" OR DE "Foster Home Care" | Expanders - Apply equivalent subjects Search modes - Boolean/Phrase | Interface - EBSCOhost Research Databases Search Screen - Advanced Search Database - APA PsycInfo | 8,816 |
| S56 | S46 OR S47 OR S48 OR S49 OR S50 OR S51 OR S52 OR S53 OR S54 OR S55 | Expanders - Apply equivalent subjects Search modes - Boolean/Phrase | Interface - EBSCOhost Research Databases Search Screen - Advanced Search Database - APA PsycInfo | 1,227,305 |
| S55 | DE ("Child" OR "Child, Preschool") | Expanders - Apply equivalent subjects Search modes - Boolean/Phrase | Interface - EBSCOhost Research Databases Search Screen - Advanced Search Database - APA PsycInfo | 194,816 |
| S54 | DE "Nursery School Students" OR DE "Nursery Schools" | Expanders - Apply equivalent subjects Search modes - Boolean/Phrase | Interface - EBSCOhost Research Databases Search Screen - Advanced Search Database - APA PsycInfo | 971 |
| S53 | DE(young person) | Expanders - Apply equivalent subjects Search modes - SmartText Searching | Interface - EBSCOhost Research Databases Search Screen - Advanced Search Database - APA PsycInfo | 20,742 |
| S52 | TI (young N2 person) OR AB ( young N2 person) | Expanders - Apply equivalent subjects Search modes - Boolean/Phrase | Interface - EBSCOhost Research Databases Search Screen - Advanced Search Database - APA PsycInfo | 3,409 |
| S51 | DE ("Child" OR "Child, Preschool") | Expanders - Apply equivalent subjects Search modes - Boolean/Phrase | Interface - EBSCOhost Research Databases Search Screen - Advanced Search Database - APA PsycInfo | 194,816 |
| S50 | AB (boy# OR boyfrien* OR boyhood* OR child* OR fifth-grader* OR first-grader* OR fourth-grader* OR girl# OR girlfriend* OR girlhood* OR juvenil * OR kid# OR kindergarten* OR minor# OR minority OR paediatric* OR peadiatric* OR pediatric* OR PICU OR preschool* OR pre-school* OR second-grader* OR seventh-grader* OR sixth-grader* OR stepchild* OR step-child* OR third-grader* OR toddler# OR young OR youngster* OR youth*) | Expanders - Apply equivalent subjects Search modes - Boolean/Phrase | Interface - EBSCOhost Research Databases Search Screen - Advanced Search Database - APA PsycInfo | 1,053,707 |
| S49 | TI (boy# OR boyfrien* OR boyhood* OR child* OR fifth-grader* OR first-grader* OR fourth-grader* OR girl# OR girlfriend* OR girlhood* OR juvenil* OR kid# OR kindergarten* OR minor# OR minority OR paediatric* OR peadiatric* OR pediatric* OR PICU OR preschool* OR pre-school* OR second-grader* OR seventh-grader* OR sixth-grader* OR stepchild* OR step-child* OR third-grader* OR toddler # OR young OR youngster* OR youth*) | Expanders - Apply equivalent subjects Search modes - Boolean/Phrase | Interface - EBSCOhost Research Databases Search Screen - Advanced Search Database - APA PsycInfo | 540,952 |
| S48 | DE ("Infant, Newborn" OR " Infant, Postmature" OR "Infant, Premature" OR "Infant") | Expanders - Apply equivalent subjects Search modes - Boolean/Phrase | Interface - EBSCOhost Research Databases Search Screen - Advanced Search Database - APA PsycInfo | 47,792 |
| S47 | AB (babies OR baby OR infan* OR neonat* OR neo-nat* OR newborn* OR new -born* OR perinat*) | Expanders - Apply equivalent subjects Search modes - Boolean/Phrase | Interface - EBSCOhost Research Databases Search Screen - Advanced Search Database - APA PsycInfo | 130,238 |
| S46 | TI (babies OR baby OR infan* OR neonat* OR neo-nat* OR newborn* OR new-born* OR perinat*) | Expanders - Apply equivalent subjects Search modes - Boolean/Phrase | Interface - EBSCOhost Research Databases Search Screen - Advanced Search Database - APA PsycInfo | 59,166 |
| S45 | S10 AND S32 AND S44 | Expanders - Apply equivalent subjects Search modes - Boolean/Phrase | Interface - EBSCOhost Research Databases Search Screen - Advanced Search Database - APA PsycInfo | 416 |
| S44 | S33 or S34 or S35 or S36 or S37 or S38 or S39 or S40 or S41 or S42 or S43 | Expanders - Apply equivalent subjects Search modes - Boolean/Phrase | Interface - EBSCOhost Research Databases Search Screen - Advanced Search Database - APA PsycInfo | 163,208 |
| S43 | TX allocat* random* | Expanders - Apply equivalent subjects Search modes - Boolean/Phrase | Interface - EBSCOhost Research Databases Search Screen - Advanced Search Database - APA PsycInfo | 13,743 |
| S42 | (MH "Quantitative Studies") | Expanders - Apply equivalent subjects Search modes - Boolean/Phrase | Interface - EBSCOhost Research Databases Search Screen - Advanced Search Database - APA PsycInfo | 512 |
| S41 | (MH "Placebos") | Expanders - Apply equivalent subjects Search modes - Boolean/Phrase | Interface - EBSCOhost Research Databases Search Screen - Advanced Search Database - APA PsycInfo | 2 |
| S40 | TX placebo* | Expanders - Apply equivalent subjects Search modes - Boolean/Phrase | Interface - EBSCOhost Research Databases Search Screen - Advanced Search Database - APA PsycInfo | 45,493 |
| S39 | TX random* allocat* | Expanders - Apply equivalent subjects Search modes - Boolean/Phrase | Interface - EBSCOhost Research Databases Search Screen - Advanced Search Database - APA PsycInfo | 13,743 |
| S38 | (MH "Random Assignment") | Expanders - Apply equivalent subjects Search modes - Boolean/Phrase | Interface - EBSCOhost Research Databases Search Screen - Advanced Search Database - APA PsycInfo | 42 |
| S37 | TX randomi* control* trial* | Expanders - Apply equivalent subjects Search modes - Boolean/Phrase | Interface - EBSCOhost Research Databases Search Screen - Advanced Search Database - APA PsycInfo | 59,247 |
| S36 | TX ( (singl* n1 blind*) or (singl* n1 mask*) ) or TX ( (doubl* n1 blind*) or (doubl* n1 mask*) ) or TX ( (tripl* n1 blind*) or (tripl* n1 mask*) ) or TX ( (trebl* n1 blind*) or (trebl* n1 mask*) ) | Expanders - Apply equivalent subjects Search modes - Boolean/Phrase | Interface - EBSCOhost Research Databases Search Screen - Advanced Search Database - APA PsycInfo | 37,025 |
| S35 | TX clinic* n1 trial* | Expanders - Apply equivalent subjects Search modes - Boolean/Phrase | Interface - EBSCOhost Research Databases Search Screen - Advanced Search Database - APA PsycInfo | 84,415 |
| S34 | PT Clinical trial | Expanders - Apply equivalent subjects Search modes - Boolean/Phrase | Interface - EBSCOhost Research Databases Search Screen - Advanced Search Database - APA PsycInfo | 1,042 |
| S33 | MH "Clinical Trials+" | Expanders - Apply equivalent subjects Search modes - Boolean/Phrase | Interface - EBSCOhost Research Databases Search Screen - Advanced Search Database - APA PsycInfo | 517 |
| S32 | S11 OR S12 OR S13 OR S14 OR S15 OR S16 OR S17 OR S18 OR S19 OR S20 OR S21 OR S22 OR S23 OR S24 OR S25 OR S26 OR S27 OR S28 OR S29 OR S30 OR S31 | Expanders - Apply equivalent subjects Search modes - Boolean/Phrase | Interface - EBSCOhost Research Databases Search Screen - Advanced Search Database - APA PsycInfo | 24,200 |
| S31 | TI (relation or relative) N3 care$ OR AB (relation or relative) N3 care$ | Expanders - Apply equivalent subjects Search modes - Boolean/Phrase | Interface - EBSCOhost Research Databases Search Screen - Advanced Search Database - APA PsycInfo | 3,720 |
| S30 | TI alternat* N3 care$ OR AB alternat* N3 care$ | Expanders - Apply equivalent subjects Search modes - Boolean/Phrase | Interface - EBSCOhost Research Databases Search Screen - Advanced Search Database - APA PsycInfo | 1,929 |
| S29 | TI "out of home" N3 care OR AB "out of home" N3 care | Expanders - Apply equivalent subjects Search modes - Boolean/Phrase | Interface - EBSCOhost Research Databases Search Screen - Advanced Search Database - APA PsycInfo | 1,447 |
| S28 | TI "out of home" N3 placement OR AB "out of home" N3 placement | Expanders - Apply equivalent subjects Search modes - Boolean/Phrase | Interface - EBSCOhost Research Databases Search Screen - Advanced Search Database - APA PsycInfo | 1,104 |
| S27 | TI "child placement" OR AB local authority N3 care$ | Expanders - Apply equivalent subjects Search modes - Boolean/Phrase | Interface - EBSCOhost Research Databases Search Screen - Advanced Search Database - APA PsycInfo | 279 |
| S26 | TI local authority N3 care$ OR AB local authority N3 care$ | Expanders - Apply equivalent subjects Search modes - Boolean/Phrase | Interface - EBSCOhost Research Databases Search Screen - Advanced Search Database - APA PsycInfo | 221 |
| S25 | TI guardian* OR AB guardian* | Expanders - Apply equivalent subjects Search modes - Boolean/Phrase | Interface - EBSCOhost Research Databases Search Screen - Advanced Search Database - APA PsycInfo | 5,282 |
| S24 | TI "living in care" OR AB "living in care" | Expanders - Apply equivalent subjects Search modes - Boolean/Phrase | Interface - EBSCOhost Research Databases Search Screen - Advanced Search Database - APA PsycInfo | 300 |
| S23 | TI ( (kin or kinship or kindred) N3 child$ ) OR AB ( (kin or kinship or kindred) N3 child$ ) | Expanders - Apply equivalent subjects Search modes - Boolean/Phrase | Interface - EBSCOhost Research Databases Search Screen - Advanced Search Database - APA PsycInfo | 610 |
| S22 | TI ( (kin or kinship or kindred) N3 father$ ) OR AB ( (kin or kinship or kindred) N3 father$ ) | Expanders - Apply equivalent subjects Search modes - Boolean/Phrase | Interface - EBSCOhost Research Databases Search Screen - Advanced Search Database - APA PsycInfo | 64 |
| S21 | TI ( (kin or kinship or kindred) N3 mother$ ) OR AB ( (kin or kinship or kindred) N3 mother$ ) | Expanders - Apply equivalent subjects Search modes - Boolean/Phrase | Interface - EBSCOhost Research Databases Search Screen - Advanced Search Database - APA PsycInfo | 137 |
| S20 | TI ( (kin or kinship or kindred) N3 parent$ ) OR AB ( (kin or kinship or kindred) N3 parent$ ) | Expanders - Apply equivalent subjects Search modes - Boolean/Phrase | Interface - EBSCOhost Research Databases Search Screen - Advanced Search Database - APA PsycInfo | 275 |
| S19 | TI ( (kin or kinship or kindred) N3 care$ ) OR AB ( (kin or kinship or kindred) N3 care$ ) | Expanders - Apply equivalent subjects Search modes - Boolean/Phrase | Interface - EBSCOhost Research Databases Search Screen - Advanced Search Database - APA PsycInfo | 858 |
| S18 | TI foster N3 child$ OR AB foster N3 child$ | Expanders - Apply equivalent subjects Search modes - Boolean/Phrase | Interface - EBSCOhost Research Databases Search Screen - Advanced Search Database - APA PsycInfo | 4,841 |
| S17 | TI foster N3 father$ OR AB foster N3 father$ | Expanders - Apply equivalent subjects Search modes - Boolean/Phrase | Interface - EBSCOhost Research Databases Search Screen - Advanced Search Database - APA PsycInfo | 152 |
| S16 | TI foster N3 mother$ OR AB foster N3 mother$ | Expanders - Apply equivalent subjects Search modes - Boolean/Phrase | Interface - EBSCOhost Research Databases Search Screen - Advanced Search Database - APA PsycInfo | 563 |
| S15 | TI foster N3 parent$ OR AB foster N3 parent$ | Expanders - Apply equivalent subjects Search modes - Boolean/Phrase | Interface - EBSCOhost Research Databases Search Screen - Advanced Search Database - APA PsycInfo | 2,603 |
| S14 | TI foster N3 care$ OR AB foster N3 care$ | Expanders - Apply equivalent subjects Search modes - Boolean/Phrase | Interface - EBSCOhost Research Databases Search Screen - Advanced Search Database - APA PsycInfo | 7,364 |
| S13 | TI foster care OR AB foster care | Expanders - Apply equivalent subjects Search modes - Boolean/Phrase | Interface - EBSCOhost Research Databases Search Screen - Advanced Search Database - APA PsycInfo | 7,678 |
| S12 | TI foster-care* OR AB foster-care* | Expanders - Apply equivalent subjects Search modes - Boolean/Phrase | Interface - EBSCOhost Research Databases Search Screen - Advanced Search Database - APA PsycInfo | 6,916 |
| S11 | (MH "Foster Home Care") OR (MH "Foster Parents") OR (MH "Child, Foster") | Expanders - Apply equivalent subjects Search modes - Boolean/Phrase | Interface - EBSCOhost Research Databases Search Screen - Advanced Search Database - APA PsycInfo | 959 |
| S10 | S1 OR S2 OR S3 OR S4 OR S5 OR S6 OR S7 OR S8 OR S9 | Expanders - Apply equivalent subjects Search modes - Boolean/Phrase | Interface - EBSCOhost Research Databases Search Screen - Advanced Search Database - APA PsycInfo | 1,193,785 |
| S9 | (MH "Schools, Nursery") | Expanders - Apply equivalent subjects Search modes - Boolean/Phrase | Interface - EBSCOhost Research Databases Search Screen - Advanced Search Database - APA PsycInfo | 1,247 |
| S8 | MH(young person) | Expanders - Apply equivalent subjects Search modes - SmartText Searching | Interface - EBSCOhost Research Databases Search Screen - Advanced Search Database - APA PsycInfo | 255 |
| S7 | TI (young N2 (person)) OR AB ( young N2 (person)) | Expanders - Apply equivalent subjects Search modes - Boolean/Phrase | Interface - EBSCOhost Research Databases Search Screen - Advanced Search Database - APA PsycInfo | 3,409 |
| S6 | MH ("Child" OR "Child, Preschool") | Expanders - Apply equivalent subjects Search modes - Boolean/Phrase | Interface - EBSCOhost Research Databases Search Screen - Advanced Search Database - APA PsycInfo | 45 |
| S5 | AB (boy# OR boyfrien* OR boyhood* OR child* OR fifth-grader* OR first-grader* OR fourth-grader* OR girl# OR girlfriend* OR girlhood* OR juvenil * OR kid# OR kindergarten* OR minor# OR minority OR paediatric* OR peadiatric* OR pediatric* OR PICU OR preschool* OR pre-school* OR second-grader* OR seventh-grader* OR sixth-grader* OR stepchild* OR step-child* OR third-grader* OR toddler# OR young OR youngster* OR youth*) | Expanders - Apply equivalent subjects Search modes - Boolean/Phrase | Interface - EBSCOhost Research Databases Search Screen - Advanced Search Database - APA PsycInfo | 1,053,707 |
| S4 | TI (boy# OR boyfrien* OR boyhood* OR child* OR fifth-grader* OR first-grader* OR fourth-grader* OR girl# OR girlfriend* OR girlhood* OR juvenil* OR kid# OR kindergarten* OR minor# OR minority OR paediatric* OR peadiatric* OR pediatric* OR PICU OR preschool* OR pre-school* OR second-grader* OR seventh-grader* OR sixth-grader* OR stepchild* OR step-child* OR third-grader* OR toddler # OR young OR youngster* OR youth*) | Expanders - Apply equivalent subjects Search modes - Boolean/Phrase | Interface - EBSCOhost Research Databases Search Screen - Advanced Search Database - APA PsycInfo | 540,952 |
| S3 | MH ("Infant, Newborn" OR "Infant") | Expanders - Apply equivalent subjects Search modes - Boolean/Phrase | Interface - EBSCOhost Research Databases Search Screen - Advanced Search Database - APA PsycInfo | 41 |
| S2 | AB (babies OR baby OR infan* OR neonat* OR neo-nat* OR newborn* OR new -born* OR perinat*) | Expanders - Apply equivalent subjects Search modes - Boolean/Phrase | Interface - EBSCOhost Research Databases Search Screen - Advanced Search Database - APA PsycInfo | 130,238 |
| S1 | TI (babies OR baby OR infan* OR neonat* OR neo-nat* OR newborn* OR new-born* OR perinat*) | Expanders - Apply equivalent subjects Search modes - Boolean/Phrase | Interface - EBSCOhost Research Databases Search Screen - Advanced Search Database - APA PsycInfo | 59,166 |

Cochrane (23062023)

Search Name:

Date Run: 23/06/2023 13:36:32

Comment:

ID Search Hits

#1 MeSH descriptor: [Foster Home Care] this term only 179

#2 MeSH descriptor: [Child, Foster] this term only 13

#3 (foster-care* OR foster care OR foster NEAR care OR foster NEAR parent OR foster NEAR mother OR foster NEAR father OR foster NEAR child):ti,ab,kw (Word variations have been searched) 1660

#4 (kin NEAR care OR kinship NEAR care OR kindred NEAR care OR kin NEAR parent OR kinship NEAR parent OR kindred NEAR parent OR kin NEAR mother OR kinship NEAR mother OR kindred NEAR mother OR kin NEAR father OR kinship NEAR father OR kindred NEAR father kin NEAR child OR kinship NEAR child OR kindred NEAR child):ti,ab,kw (Word variations have been searched) 89

#5 ("living in care" OR guardian OR "local authority" NEAR care OR child NEXT placement OR "out of home" NEAR placement OR "out of home" NEAR care OR alternative NEAR care):ti,ab,kw (Word variations have been searched) 7285

#6 ("living in care"):ti,ab,kw OR (guardian):ti,ab,kw OR ("local authority" NEAR care):ti,ab,kw OR (child NEXT placement):ti,ab,kw OR ("out of home" NEAR placement):ti,ab,kw (Word variations have been searched) 6058

#7 ("out of home" NEAR care):ti,ab,kw OR (alternative NEAR care):ti,ab,kw (Word variations have been searched) 1243

#8 #1 OR #2 OR #3 OR #4 OR #5 OR #6 OR #7 8909

#9 (boy*):ti,ab,kw OR (boyfrien*):ti,ab,kw OR (boyhood*):ti,ab,kw OR (child*):ti,ab,kw AND (fifth-grader*):ti,ab,kw (Word variations have been searched) 8180

#10 (first-grader*):ti,ab,kw OR (fourth-grader*):ti,ab,kw OR (girl*):ti,ab,kw OR (OR girlfriend*):ti,ab,kw OR (OR girlhood*):ti,ab,kw (Word variations have been searched) 8957

#11 (juvenil *):ti,ab,kw OR (kid):ti,ab,kw OR (kindergarten*):ti,ab,kw OR (minor):ti,ab,kw OR (minority):ti,ab,kw (Word variations have been searched) 27647

#12 (paediatric*):ti,ab,kw OR (peadiatric*):ti,ab,kw OR (pediatric*):ti,ab,kw OR (PICU):ti,ab,kw OR (preschool*):ti,ab,kw (Word variations have been searched) 78373

#13 (pre-school*):ti,ab,kw OR (second-grader*):ti,ab,kw OR (seventh-grader*):ti,ab,kw OR (sixth-grader*):ti,ab,kw OR (stepchild*):ti,ab,kw (Word variations have been searched) 1010

#14 (step-child*):ti,ab,kw OR (third-grader*):ti,ab,kw OR (toddler#):ti,ab,kw OR (OR young):ti,ab,kw OR (youngster*):ti,ab,kw (Word variations have been searched) 2395

#15 (youth):ti,ab,kw (Word variations have been searched) 9736

#16 (babies OR baby OR infan* OR neonat* OR neo-nat* OR newborn* OR new-born* OR perinat*):ti,ab,kw 93954

#17 (young NEAR person):ti,ab,kw 731

#18 MeSH descriptor: [Infant] this term only 28623

#19 MeSH descriptor: [Infant, Newborn] this term only 20279

#20 MeSH descriptor: [Child] this term only 69223

#21 MeSH descriptor: [Child, Preschool] this term only 35097

#22 MeSH descriptor: [Nurseries, Infant] explode all trees 13

#23 #9 OR #10 OR #11 OR #12 OR #13 OR #14 OR #15 OR #16 OR #17 OR #18 OR #19 OR #20 OR #21 OR #22 214116

#24 MeSH descriptor: [Clinical Trial] explode all trees 45348

#25 (Clinical Trial):ti,ab,kw 723656

#26 (Clinic* NEAR trial*):ti,ab,kw 543306

#27 (singl* NEAR blind*):ti,ab,kw OR (singl* NEAR mask):ti,ab,kw OR (doubl* NEAR blind*):ti,ab,kw OR (doubl* NEAR mask*):ti,ab,kw (Word variations have been searched) 396643

#28 (tripl* NEAR blind*):ti,ab,kw OR (tripl* NEAR mask*):ti,ab,kw OR (trebl* NEAR blind*):ti,ab,kw OR (trebl* NEAR mask*):ti,ab,kw (Word variations have been searched) 2918

#29 (randomis* control* trial*):ti,ab,kw (Word variations have been searched) 124429

#30 MeSH descriptor: [Random Allocation] this term only 23362

#31 (placebo*):ti,ab,kw (Word variations have been searched) 366717

#32 MeSH descriptor: [Placebos] this term only 25631

#33 (allocat* NEAR random*):ti,ab,kw (Word variations have been searched) 84684

#34 #24 OR #25 OR #26 OR #27 OR #28 OR #29 OR #30 OR #31 OR #32 OR #33 1088589

#35 #8 AND #23 AND #34 2056
