# Supplementary material for: Interventions for pre‐school children in foster care: A systematic review of randomised controlled trials of child‐related outcomes
Source: JCPP Adv. 2024 Sep 5;5(1):e12273. doi: 10.1002/jcv2.12273 (PMC11889642; doi:10.1002/jcv2.12273)
Supplement: Supplementary file 2 — Supporting Information S2 [file JCV2-5-e12273-s002.docx]

| **Study + country** | **Demographics of included participants** | **Sample Size** | **Intervention description** | **Intervention focus** | **Control Group** | **Main outcomes + measures** | **Outcome timing** | **Findings** |
| --- | --- | --- | --- | --- | --- | --- | --- | --- |
| Bernard et al., 2017  USA | Child: Mean age at follow-up 39.52 months (SD = 2.98, range 34.2-46.4 months) 44% male African American 56%, Asian American 2%, Biracial 8%, Caucasian 29%, Hispanic 6%  Caregiver:  Mean age 43.2 years (SD = 9.76, range 19-58.6 years) 94% female  African American 48%, Biracial 4%, Caucasian 44%, Hispanic 6% | 52 infants and foster caregivers  Intervention n=24  Control n=28 | **Attachment and Biobehavioural Catch-Up (ABC)**  10 weekly 1-hr sessions  Delivered at participant's home  Manualised format  Delivered by parent trainers  Videotaped | Dyadic  Caregiver behaviours  Sensitivity  Responsiveness  Nurturance | **Developmental Education for Families (DEF)** 10 weekly 1-hr sessions, 1-hour each, video recorded, at participant's home.  Focus on cognitive and linguistic development | Receptive language - Peabody Picture Vocabulary Test, third edition | 2 years post intervention | Infants who received the ABC intervention scored significantly higher on receptive vocabulary at age 36 months than those who received a control intervention |
| Dozier et al., 2009  USA | Child: Mean age at follow-up assessment 18.9 months (range 3.9-39.4 months) 50% male African American 63%, White Non-Hispanic 26%, Hispanic 4%, Biracial 7%  Caregiver: 91% female | 46 children and foster caregivers  Intervention n=22  Control n=24 | **Attachment and Biobehavioural Catch-Up (ABC)**  As above | As above | **Developmental Education for Families (DEF)**  As above | Attachment -  Parent Attachment Diaries  Strange Situation Procedure | 1 month post intervention | Children who received the ABC intervention showed significantly less avoidance than those received a control intervention |
| Dozier et al., 2006  USA | Child: Age range 3.6-39.4 months 50% male African American 63%, White 32%, Biracial 5%  Caregiver: 93% female | 60 children and foster parents | **Attachment and Biobehavioural Catch-Up (ABC)**  As above | As above | **Developmental Education for Families (DEF)**  As above | Saliva samples and cortisol assay  Problem Behaviours - Parent Daily Report | 1 month post intervention | Children in the ABC group had lower cortisol values than children in the control group, and parents in the intervention group reported fewer behaviour problems for older children compared to younger foster children |
| Lewis-Morrarty et al., 2012  USA | Child: Mean age 60.3 months (SD=8.6, range 4-6 years) 50.8% male 42.6% African American, 36.1% European American, 21.3% Hispanic, Asian American, or Biracial  Parent: All female  39.3% were African American, 57.4% European American, 3.3% were Asian American | 61 children and foster carers  Intervention n=17  Control n=20 | **Attachment and Biobehavioural Catch-Up (ABC)**  As above | As above | **Developmental Education for Families (DEF)**  As above | Theory of Mind - Penny-hiding game  Receptive Language Abilities - Peadbody Picture Vocabulary Test-Third Edition  Cognitive Flexibility - Dimensional Change Card Sort | Children assessed annually until the age of 6 years | Children who received ABC showed stronger cognitive flexibility and theory of mind skills, compared to those who received the control intervention |
| Lind et al., 2017  USA | Child: Age range 14-56 months  Gender 53% male White 26%, African American 56%, Asian American 1%, Biracial 11%  Caregiver: Age range 22-76 years 95% female White 44%, African American 46%, Hispanic 4%, Biracial 5% | 173 parent-toddler dyads   Intervention n=63 toddlers, n=51 parents  Control n=58 toddlers, n=48 parents | **Attachment and Biobehavioural Catch-Up for Toddlers (ABC-T)** As for ABC  ABC-T also teaches strategies for parents to be coregulators for children when they become dysregulated | Dyadic  Caregiver behaviours  Sensitivity  Responsiveness  Nurturance  Co-regulation | **Developmental Education for Families (DEF)**  As above | Child Executive Functioning - Child Behaviour Checklist, attention problems scale; Dimensional Change Care Sort | Post intervention when child aged 48 months | Foster children whose parents received ABC-T had fewer parent-reported attention problems and demonstrated greater cognitive flexibility than foster children whose parents received the control intervention |
| Raby et al., 2019  USA | Child: Intervention –  Mean age 52.1 months (SD=9.1)  46.7% female  31.1% Caucasian, 46.7% African American, 8.9% Hispanic, 13.3% Other  Control –  Mean age 51.4 months (SD=8.7) 51.2% female 25.6% Caucasian, 51.2% African American, 7.0% Hispanic, 16.3% Other  Caregiver: Intervention - age 47 years  86.7% female  50% Caucasian, 43.2% African American, 4.5% Hispanic, 2.3% Other  Control - age 48 years 97.7% female 33.3% Caucasian, 50% African American, 7.1% Hispanic, 9.5% Other | 88 foster children and caregivers  Intervention n=45  Control n=43 | **Attachment and Biobehavioural Catch-Up for Toddlers (ABC-T)**  As above | As above | **Developmental Education for Families (DEF)**  As above | Receptive vocabulary skills - Peabody Picture Vocabulary Test, third edition | Post intervention when child aged between 36-60 months old | Children whose foster parents received ABC-T demonstrated more advanced receptive vocabulary abilities than children whose foster parents received the control intervention. The positive effect of ABC-T on foster children’s receptive vocabulary was mediated by increases in foster parents’ sensitivity during parent-child interactions. |
| Fisher & Kim, 2007  USA | Child: Mean age at baseline 4.4 years (range = 4.3–4.5 years) Intervention 49% male Control 58% male 89% European American, 5% Latino, 5% Native American, 1% African American  Parent: Not reported | 117 foster children and caregivers  Intervention n=57  Control n=60 | **Multidimensional Treatment Foster Care for Preschoolers (MTFC-P) intervention**  Duration 6-9 months  Includes foster children, foster caregivers, and birthparents or adoptive families  12 hours of foster-parent training prior to placement + weekly group meetings + phone support  Children receive individualised treatment with therapists and weekly therapeutic playgroup session | Dyadic  Preventative intervention  Designed to address developmental and social-emotional needs of pre-school children  Child self-regulation  Caregiver responsiveness + consistency | **Regular Foster Care (RFC)**  Care as usual.  Typically included at least monthly contact with caseworkers (may lead to weekly individual psychotherapy, medication or special education services) | Attachment-related behaviours - Parent Attachment Diary | Post-intervention at 3-month intervals up to 12 months | Children in the intervention group showed significant increases in secure behaviour and decreases in avoidant behaviour compared to children in the control group. Both groups showed significant decreases in resistant behaviour over time. Analyses also revealed a significant interaction between treatment condition and age at first foster placement on change in secure behaviour. |
| Fisher et al., 2011  USA | Child: Mean age at baseline 4.4 years (range = 4.3–4.5 years) Intervention 49% male Control 58% male 89% European American, 5% Latino, 5% Native American, 1% African American  Parent: Not reported | 117 foster children and caregivers  Intervention n=57 Control n=60 | **Multidimensional Treatment Foster Care for Preschoolers (MTFC-P) intervention** As above | As above | **Regular Foster Care (RFC)**  As above | Child problem behaviour - Parent Daily Report, via telephone | Monthly for 3 months (6 observations) | Foster care children with 5 or fewer problem behaviour were at low risk for disruption, but risk increased 10% for each additional behaviour . The intervention appeared to mitigate this. |
| Bruce et al., 2009  USA | Child: Intervention: Mean age 6.08 years (SD=0.57) Males n=6   Control: Mean age 5.92 years (SD=0.68) Males n=6 | n=34  Intervention n=10  Control n=13 | **Multidimensional Treatment Foster Care for Preschoolers (MTFC-P) intervention**  As above | As above | **Regular Foster Care (RFC)**  As above | Flanker Task - cognitive control and response monitoring  EEG recording and analysis - electrophysiological measures of response monitoring | Not provided | No group differences on the behaviour al measures of cognitive control or response monitoring. |
| Jonkman et al., 2017  Netherlands | Child:  64% male Mean age 63.51 months Age range 3-7 years | 34  Intervention n=23  Control n=11 | **Multidimensional Treatment Foster Care for Preschoolers (MTFC-P)**  As above | As above | **Treatment Foster Care As Usual** Phase 1 - diagnostic screening phase immediately after referral, via home visits and contact with psychologist or psychiatrist Phase 2 - treatment adapted to needs of children and families. 2 weekly home visits where social workers coach foster parents in parental skills and provide children with support. Can arrange additional specific interventions if needed. | Behavioural and relationship functioning (CBCL, Teacher Report Form, Parent Daily Report)   Disturbances of Attachment Interview (DAI)   Trauma symptoms (Trauma Symptom Checklist for Young Children)  HPA axis (salivary cortisol) | Every 3 months through duration of treatment; baseline, 3, 6, 9 months | In the Netherlands, MTFC-P was not superior to treatment foster care as usual in treating behaviour al problems, symptoms of attachment disorder, foster parent stress, and neurobiological functioning of children and foster parents.  For the first 6 months, trauma symptoms in MTFC-P remained stable and there was a negative treatment effect for TAU, but in the last 3 months there was an advantage from TAU compared to MTFC-P. This was substantial enough a decrease in trauma symptoms which led to overall treatment effect in favour of TAU Positive effects seen in USA studies - may be related to different FC systems, and differences between what is TAU in each country |
| Pears et al., 2012  USA | Child: Intervention: Mean age = 5.26 (SD 0.33) 52% male 62% non-kinship foster care Ethnicity: 55% european american, 30% latino, 1% african american, 2% native american, 2% pacific islander, 10% mixed race  Control: Mean age = 5.25 (0.35) 46% male 61% non-kinship foster care Ethnicity: 51% european american, 31% latino, 0% african american, 0% native american, 0% pacific islander, 18% mixed race | N=192  Intervention n=102  Control n=90 | **Kids in Transition to School (KITS)**  16-week group based school readiness curriculum  Delivered to children + groups for caregivers  24 x 2-hr child sessions twice weekly in the summer + 2-hr sessions once weekly in the fall  8 x 2-hr caregiver groups fortnightly   School readiness phase + transition / maintenance phase | To increase school  readiness and promote better school functioning | **Foster Care Comparison Group (FCC)**  Service as usual | Oppositional and aggressive classroom behaviours - Teacher Report Form aggressive + delinquent subscales  Oppositional subscale of Conners' Teacher Ratings Scales-Revised | Up to 8 months post-intervention | The KITS Program appears to decrease the likelihood that the children will be oppositional and aggressive in their classrooms. |
| Pears et al., 2013  USA | Child: Intervention: Mean age = 5.26 (0.33) 52% male 62% non-kinship foster care Ethnicity: 55% european american, 30% latino, 1% african american, 2% native american, 2% pacific islander, 10% mixed race  Control: Mean age = 5.25 (0.35) 46% male 61% non-kinship foster care Ethnicity: 51% european american, 31% latino, 0% african american, 0% native american, 0% pacific islander, 18% mixed race  Caregivers: not provided | N=192  Intervention n=102  Control n=90 | **Kids in Transition to School (KITS)**  As above | As above | **Foster Care Comparison Group (FCC)**  As above | Early Literacy Skills - Letter Naming Fluency + Sound Fluency subtests of the Dynamic Indicators of Basic Early Literacy Skills; 24-item Concepts About Print Test; caregiver rating of prereading skills  Prosocial Skills - Preschool Penn Interactive Peer Play Scale: Play Interaction; Play Disruption and Play Disconnection subscales; child completed vignette task  Regulation -  Inhibitory control - Children’s Behaviour Questionnaire; Brief Rating Inventory of Executive Function–Preschool Version (BRIEF-P); Child Behaviour Checklist; Emotion Regulation Checklist | 4 months post-intervention | The intervention had significant positive effects on early literacy and self-regulatory skills. |
| Pears et al., 2016  USA | Child: Intervention: Mean age = 5.26 (0.33) 52% male 62% non-kinship foster care Ethnicity: 55% european american, 30% latino, 1% african american, 2% native american, 2% pacific islander, 10% mixed race  Control: Mean age = 5.25 (0.35) 46% male 61% non-kinship foster care Ethnicity: 51% european american, 31% latino, 0% african american, 0% native american, 0% pacific islander, 18% mixed race  Caregivers:  Not provided | N=192  Intervention n=102 Control n=90 | **Kids in Transition to School (KITS)**  As above | As above | **Foster Care Comparison Group (FCC)**  As above | Positive attitudes towards alcohol use in third grade - adapted from Monitoring the Future National Survey Questionnaire  Positive attitudes towards antisocial behaviour in third grade - bespoke  Involvement with deviant peers in third grade - child and teacher bespoke  Self-competence in third grade - Global Self-Worth Scale of the Self-Perception Profile for Children | Post intervention age 5 + 6 years + subsequent school years up to 9 years old | The intervention decreased positive attitudes towards alcohol use and antisocial behaviours and positively influenced children’s third-grade self-competence, which in turn, decreased their involvement with deviant peers. |
| Mersky et al., 2016  USA | Child: Mean age 4.6 years 54% female 70% racial and ethnic minorities (61% African American)   Foster carer: 89% female 51% racial and ethnic minorities 52% married | 102 dyads   Brief PCIT n=39  Extended PCIT n=19  Waitlist control n=33 | **Parent Child Interaction Therapy (PCIT)**  2 phases: Child-Directed Interaction (CDI) + Parent-Directed Interaction (PDI)  Coaching by practitioners  Individual sessions (typically 12-20 sessions) + group foster parent training  Brief condition – 2xdays training and 8 weeks phone calls  Extended condition – 2xdays training, 14 weeks phone calls + booster training day | Dyadic behavioural intervention  Management of child behaviour using authoritative parenting approach  Parenting skills  Caregiver child relationships | **Waitlist Control**  Services as usual | Eyberg Child Behaviour Inventory Child Behaviour Checklist | 8 + 14 weeks | Compared to controls, children in both PCIT groups exhibited a greater reduction in externalizing and internalizing scores over time. |
| Danko, 2014  USA | Child:  74.1% male Mean age 3.56 years (SD=0.99, range 2.08-5.67) Ethnicity 66.7% African American, 11.1% Latino, 3.7% Caucasian, 3.7% Asian, 14.8% Multiracial  Foster parent:  92.6% female Mean age 44.11 years (SD=13.12 range 23-81) Ethnicity 66.7% African American, 14.8% Latino, 18.5% Caucasian | 24 families  CDI-only PCIT  n=8  CDI plus PDI PCIT n=7  Waitlist bibliotherapy control n=9 | **CDI (Child-Directed Interaction) only**  10-14x60-minute CDI sessions twice a week  **CDI plus PDI (Parent-Directed Interaction)** 5-7xCDI and 5-7xPDI sessions twice a week for 10-14 sessions of 60-minutes.   Therapist coaching, feedback + homework  Requirement to reach mastery before moving to PDI.  Delivered by PI + 2 doctoral students with 1+ years of training in PCIT | As above | **Waitlist bibliotherapy** Educational handouts with written description of the CDI and PDI phase skills + additional parenting “tips sheets” | Eyberg Child Behaviour Inventory - Intensity Scale measuring frequency of disruptive behaviours  Child Behaviour Checklist   Beck Depression Inventory II  Parenting Stress Index Short Form  Therapy Attitude Inventory  Attachment Q-Set | Immediately post intervention | No significant difference between families receiving PCIT and families receiving waitlist bibliotherapy on post-study attachment security.  Children in CDI plus PDI had a more secure relationship with their foster parent at post-treatment than CDI only No significant difference between PCIT and waitlist on post-study parenting stress or post-study depression |
| N'zi et al., 2016  USA | Child:  50% female Mean age 5.2 years (range 2.0-7.5) Ethnicity 64% caucasian, 22% African American, 7% Hispanic, 7% biracial  Caregivers:  86% grandmothers, 14% great-grandmothers, mean age 56.5 years (range 45.9-73.0) Mean length of placement 3.01 years (range 3 months - 7.5 years) 14% of children adopted, 29% in permanent guardianship, 43% temporary guardianship, 14% informal guardianship arrangements (outside of court or CWS involvement) | 14 dyads Intervention n=7 Control n=7 | **Child Directed Interaction Training (CDIT)** Twice weekly 8-session. Delivered at a local community library by advanced graduate student trainers. Weekly case supervision. Followed standard PDIT protocol - skills in first session / 7 coaching sessions / practicing skills with trainer coaching | As above | **Waitlist Control**  Service as usual | Eyberg Child Behaviour Inventory  Child Behaviour Checklist  Child-Parent Relationship Scale   Dyadic Parent-Child Interaction Coding System  Parent Daily Report and Daily Discipline Inventory | Post CDIT training, before treatment for waitlist group, approx. 7 weeks post baseline,   3 months post intervention for CDIT group only. | CDIT group demonstrated more positive relationships with their children and fewer externalizing child behaviour problems than waitlist. Results appeared stable at 3-month follow-up. |
| Conn et al., 2018  USA | Child: Intervention: Mean age 53.33 months (SD=16.81) 40% female  Control: Mean age 42.88 months (SD=12.59) 23.5% female  Caregiver: Intervention: 81.3% female 25% aged 18-35, 62.5% aged 36-44, 12.5% aged over 45  Control: 93.8% female 29.4% aged 18-35, 58.8% aged 36-44, 11.8% aged over 45 | N=33  Intervention n=16  Control n=17 | **Incredible Years (IY)**  2.5-hr weekly sessions, weekly for 13 weeks  Delivered to caregivers in group format  Community-based location  Parent groups led by Masters-level psychologist certified in IY curriculum | Trauma informed version of IY  Includes specific information on childhood trauma  Positive parenting + engaging with child  Parenting skills, responsiveness, nurturance | **Treatment As Usual** | Child behaviour - Child Behaviour Checklist | Post intervention 13 weeks | No between group differences were found for measures of child behaviour. |
| Job et al., 2020  Germany | Child: Intervention: Mean age 42.8 months (SD=18.1, range 24-91) 43% female Mean duration of stay in current foster family 17.3 months (SD=8.3, range 3-25)  Control:  Mean age 50.6 months (SD=19.8, range 24-95) 54% female Mean duration of stay in current foster family 18.2 months (SD=8.5, range 2-40)  Carers: Intervention: Mothers' mean age 40.4 years (SD=7.1, range 25-57) Fathers' mean age 44.8 years (SD=6.6, range 32-58)  Control: Mothers' mean age 43 years (SD=6.2, range 29-56) Fathers' mean age 45.4 years (SD=6.7, range 34-62) | 81 foster families, 87 foster children  Intervention n=44 families, 46 foster children Control n=37 families, 41 foster children | **Triple P system for foster parents (TCTP)**  Five 2.5-hr weekly group sessions + two 20-min telephone consultations + group closure session.  Facilitators experienced in the Triple P model were trained in specific approach.  Group sessions video recorded to allow for assessment of fidelity  Intervention mean length of 7.2 weeks (SD=2.9). | Positive parenting  Consistency  Managing behaviours | **Usual Care** | Child Relationship Development Inventory  Dyadic Parent–Child Interaction Coding System 4th edition  Mother–Child Play Task Observation System  Diagnostic Interview of Mental Disorders in Childhood and Adolescents  Eyberg Child Behaviour Inventory  Preschool Anxiety Scale | Time 1 - 6 months post baseline Time 2 - 12 months post baseline | No advantages of the intervention group compared with usual care on any outcome measure. |
| Lipscomb et al., 2013  USA | Child: 47% female  Intervention: Mean age at baseline 48.25 months (SD=6.70) Ethnicity 43% Anglo-American, 39% African- American, and 18% Hispanic-American  Control: Mean age at baseline 47.79 months (SD=7.29) Ethnicity 47% Anglo-American, 37% African-American, and 16% Hispanic- American   Carer: Non-parental primary caregiver at baseline included great-grandmother (66%), foster parent (13%), other female non-relative (10%), grandmother (4%), sister/step-sister (3%), great‐grandfather (2%), grandfather (1%), and other male non-relative (1%). | 253 children  Intervention (61%) n=154 Control (39%) n=99 | **Head Start**  Publicly financed early childhood education and care program  Comprehensive services to support disadvantaged preschool-age children and their families  Provides quality early learning, parental support, and wrap-around services | Head Start aims to promote development of the whole child  Provides a variety of services aimed at improving learning skills, social skills, and overall health of children  Regards parental involvement through family services as essential | **Community Control Group** Could be enrolled in Head Start in the second year of the study | Teacher-child relationship - total positive relationship scale of the Student–Teacher Relationship Scale  Externalizing Behaviour Problems - Achenbach Child Behaviour Checklist, Adjustment Scales for Preschool Intervention | At the end of the HeadStart year + 1 year later | Modest direct short-term and indirect longer-term impacts of Head Start on school readiness outcomes (increased pre-academic skills, more positive teacher–child relationships, and reductions in behaviour problems) in intervention group compared to control |
| Kyunghee Lee & Jung-Sook Lee, 2016  USA | Child: Intervention: Mean age 3.4 years (SD=0.5) 53% female Ethnicity 40% Black, 13% Hispanic, 46% White   Control: Mean age 3.4 years (SD=0.5)  Ethnicity 54% Black, 20% Hispanic, 26% White  Carer: Intervention: Mean age 39.5 years (SD=14.0) 17% married 44% less than high school education  Control: Mean age 34.7 years (SD=12.3) 22% married 29% less than high school education | 162 children  Intervention n=97 Control n=65 | As above | As above | **Non-Head Start Group** Could be enrolled in Head Start in the second year of the study if they remained eligible and did not go on to kindergarten | Positive child and parent relationship - total positive relationship scale based on the Robert Pianta scales  Positive child and teacher relationship - based on the Robert Pianta scales  Children’s hyperactive scores and aggressive scores - Adjustment Scales for Preschool Intervention  Social skills and positive approaches to learning - parent rating | At the end of the HeadStart year +  1 year later | Head Start enhanced social-emotional outcomes for children in foster care. The positive impacts of Head Start on children’s social-emotional outcomes were greater when parents read books frequently. |
